# Implicit-solvent dissipative particle dynamics force field based on a four-to-one coarse-grained mapping scheme

**DOI:** 10.1371/journal.pone.0198049

**Published:** 2018-05-24

**Authors:** Mingwei Wan, Lianghui Gao, Weihai Fang

**Affiliations:** Key Laboratory of Theoretical and Computational Photochemistry, Ministry of Education, College of Chemistry, Beijing Normal University, Beijing, China; Hong Kong University of Science and Technology, HONG KONG

## Abstract

A new set of efficient solvent-free dissipative particle dynamics (DPD) force fields was developed for phospholipids and peptides. To enhance transferability, this model maps around four heavy atoms and their connected hydrogen atoms into a coarse-grained elementary bead based on functional group. The effective hybrid potential between any pair of beads is composed of a short-range repulsive soft-core potential that directly adopts the form of an explicit-solvent DPD model and a long-range attractive hydrophobic potential. The parameters of the attractive potentials for lipid molecules were obtained by fitting the explicit-solvent DPD simulation of one bead of any type in a water box, then finely tuning it until the bilayer membrane properties obtained in the explicit-solvent model were matched. These parameters were further extended to amino acids according to bead type. The structural and elastic properties of bilayer membranes, free energy profiles for a lipid flip-flop and amino acid analogues translocating across the membrane, and membrane pore formation induced by antimicrobial peptides obtained from this solvent-free DPD force field considerably agreed with the explicit-solvent DPD results. Importantly, the efficiency of this method is guaranteed to accelerate the assembly of vesicles composed of several thousand lipids by up to 50-fold, rendering the experimental liposome dynamics as well as membrane-peptide interactions feasible at accessible computational expense.

## Introduction

Simulations of molecular dynamics (MD) play crucial roles in interpreting molecular interactions and assembly, particularly when they are unreachable experimentally because of limited time and length resolutions [1–3]. Examples encompass the assembly of a plethora of structures in soft matter and biological systems. In principle, all-atom (AA) MD captures the most full-scale information but provides access only to systems containing several hundred molecules on a nanosecond time scale because of the copious amount of computation [2, 3]. For actual soft matter and biological systems such as biomembrane transformations (e.g., phase transition between gel and fluid, raft formation, microtube growth, vesiculation, and membrane fusion), the scale is far beyond the abilities of AAMD [4, 5]. This hurdle between atomic resolved simulation and natural chemistry can be lessened by coarse-graining several atoms into one particle [4, 5].

The traditional explicit-solvent coarse-grained (CG) method, however, continues to lack sufficient speed for solving the majority of membrane-associated processes. In these cases, the computational burden is related to the bulk solvent and becomes heavier when the membrane deforms to a great degree or the spatial size of the simulated system increases markedly [6, 7]. In one example, in a CG simulation of a tether pulling from a membrane, the simulated system had as many as 4 million CG particles, of which more than 90% were CG water beads [6]. In another, a spherical vesicle of radius *R* was simulated. The fraction of its sheet-like membrane area (*R*^2^) in the simulation box (*L*^3^) was approximately *R*^2^/*L*^3^ ≈ 1/*R*, which decreased rapidly as a function of *R* [7]. By neglecting the solvent degrees of freedom, a significant enhancement in calculation efficiency can be achieved. With this consideration, various implicit-solvent force fields have been developed over the past decade. Milano and coworkers introduced a hybrid particle field MD scheme with compatibility across AA and/or particular CG models [8]. It was validated over various molecular models, including biomembranes [9]. The method combined a discrete representation of the molecules (AAMD or CGMD) with a continuous self-consistent field description of the inter-molecular interactions. A model such as this requires storage for all the particles’ coordinates, and it loses a direct route to introduce implicit solvent. Wang and Deserno explicitly introduced a cohesive potential to mimic the hydrophobic attractions between the lipid tails in a one-component membrane in the fluid phase [10] and applied it to other similar, but simple, CG lipids by changing the topology of the lipid [11]. The model has poor transferability to complicated lipids. This transferability issue could be significantly solved by choosing a versatile and straightforward parameterization for the potential related to the implicit solvent. In this situation, the Newton’s inversion method used by Lyubartsev et al. [12] or the force matching method employed by the Voth group [13, 14] could be useful, but they remain insufficient at this moment because of the impractical procedures used to obtain potential and/or parameters, wherein the effective CG forces were extracted from the radial distribution functions or the effective non-bonded forces between selective CG interaction sites from a reference AA simulation.

Because chemical details matter in many cases (e.g., complicated peptide-membrane interactions, local membrane structural transformation induced by mini environmental stimulus, and the chemically heterogeneous Gram-negative bacteria membrane), a sufficiently high CG resolution is required. The CG Martini model provides a force field that considers transferability and chemical specificity[6, 15–20]. This model maps about four heavy atoms and their connected hydrogen atoms into a coarse-grained elementary bead. These beads are divided by charge type, polarizability, and hydrogen-bonding capacity. Embedded in the free-source, industry-wide, and constantly updated software GROMACS [21–25], Martini was developed to investigate a large variety of biological processes. The accuracy of Martini is guaranteed by the thermodynamic data calculations for chemical building blocks, usually representative small compounds, matched with experimental values [15–20]. In addition, a broad range of applications involving phospholipids [15], proteins [16, 18], carbohydrates [17], glycolipids [19], DNA [20], etc. are realized with no reparameterization, benefitting from the extensively validated building blocks. For the sake of speed, the solvent-free version of Martini [26], nicknamed “Dry” Martini, was implemented based on the original Martini force field, inheriting the building block feature. The Dry Martini force field, however, continues to impose a severe computational requirement because of the “hard” Lennard-Jones (L-J) potential, which limits the time step used in Newton’s equation of motion in the series of GROMACS-based force fields.

Compared to the hard sphere models in most CGMD force fields, including Martini, the dissipative particle dynamics (DPD) model maps atoms into soft beads that can overlap [27–49]. These soft beads interact via short-ranged conservative, random, and dissipative forces. The soft potential increases the time step considerably, speeding up the simulation and making it easy for beads to escape local minimums formed by surrounding particles. In addition, DPD explicitly includes fluctuation and dissipation stemming from the neglected degrees of freedom. More importantly, the latest set of DPD parameters [45], updated by adopting the Martini-like four-to-one mapping scheme [15], has strengthened portability. It matches the correct water compressibility and the experimental Flory-Huggins χ parameter. Membrane properties simulated by this set of parameters are consistent with atomistic simulations [50–52] and experimental measurements [53–56].

A DPD model that includes an implicit solvent (Im-DPD) has been developed for studying ethanol-water mixtures [57], block copolymers [58, 59], and liquid drops surrounded by a gas [60]. Silbermann et al. extracted effective pairwise additive CG potentials from AAMD simulations, but which lacked in an analytic form [57]. Panagiotopoulos’s group introduced L-J type implicit potentials, which, however, were too “hard” [58, 59]. Liu and Meakin combined an analytic soft-core smoothing function with DPD to simulate binary mixtures, Poiseuille flow, and liquid drop formation [60]. However, the implicit solvent potential has not been developed and applied to biomembrane systems. Only in recent years has a solvent-free DPD model for lipid membranes been available [61, 62]. Sevink and Fraaije rigorously derived the implicit solvent potential by systematically averaging the solvent degrees of freedom via a hybrid particle-continuum method. A long-range attractive hydrophobic potential with an analytic form was obtained. Three parameters in this additional potential were tuned to match the membranes and vesicles, as simulated by DPD with an explicit solvent (Ex-DPD) [31, 38]. A 20-fold decrease in required calculational time was achieved by implicitly describing the bulk solvent. However, this implicit-solvent force field was based on a very simple CG model of lipid molecules and less transferable Ex-DPD force parameters [31, 37, 38]. Applying this to the Martini-like DPD beads with chemical specificity resulted in implicit-solvent force parameters that were difficult to tune using the methods of Ref. [61, 62]. We have developed a more direct routine for constructing the Im-DPD potentials. In our method, two parameters were obtained by fitting the explicit-solvent force acting on a CG bead immersed in a pure water box, and one was finely tuned by matching the structure of a lipid bilayer membrane. The validity of this new solvent-free force parameter set was justified by simulating essential structural and elastic properties of bilayer membrane, partition energy of a lipid molecule or amino acid analogues into a bilayer membrane, and pore formation induced by antimicrobial peptides.

## Theory of Im-DPD

In Ex-DPD simulation, for each pair of beads *i* and *j* separated by a distance *r*_*ij*_ < *r*_0_, a conservative force

$$F_{ij}^{C}(r_{ij})\;=\;a_{ij}(1-\frac{r_{ij}}{r_{0}}){\hat{r}}_{ij},$$ (1)

a dissipative force

$$F_{ij}^{D}(r_{ij})\;=\;-\gamma_{ij}{\left(1-\frac{r_{ij}}{r_{0}}\right)}^{2}({\hat{r}}_{ij}∙v_{ij}){\hat{r}}_{ij},$$ (2)

and a random force

$$F_{ij}^{R}(r_{ij})\;=\;\sqrt{2\gamma_{ij}k_{B}T}(1-\frac{r_{ij}}{r_{0}})\zeta_{ij}{\hat{r}}_{ij},$$ (3)

are combined to describe their interactions. Here, ***r***_*ij*_ = ***r***_*i*_-***r***_*j*_, *r*_*ij*_ = |***r***_*ij*_|, ***ȓ***_*ij*_ = ***r***_*ij*_/*r*_*ij*_, and *r*_0_ is the interaction cutoff. The vector ***v***_*ij*_ = ***v***_*i*_-***v***_*j*_ is the velocity difference between beads *i* and *j*. The DPD force parameter *a*_*ij*_ (in units of *k*_B_*T*/*r*_0_, where *k*_B_ is the Boltzmann constant and *T* is the temperature) represents the maximum repulsion strength. The parameter *γ*_*ij*_ represents the friction coefficient (in units of $\sqrt{k_{B}Tm_{0}/{r_{0}}^{2}}$, where *m*_0_ is the bead mass). The random force parameter *ζ*_*ij*_ is a symmetrically and uniformly distributed random number.

To average the solvent degrees of freedom, Sevink and Fraaije [61] employed the hybrid particle/continuum method where the hybrid (free) energy for particle *k* and one monomeric (solvent) field *ρ* is given by

$$E[\{r_{k}\},\rho ]\;=\;E^{p}(\left\{r_{k}\right\})+E^{f}[\rho ]+E^{pf}[\{r_{k}\},\rho ].$$ (4)

The first term is the usual potential energy in a particle description. The second term is the usual free energy for the field description. Its form is shown in

$$E^{f}[\rho ]\;=\;k_{B}T\int_{V}^{}\rho (r)[\text{ln}\;\Lambda^{3}\rho (r)-1]dr+\frac{\kappa_{H}}{2}\int_{V}^{}{\rho (r)}^{2}dr,$$ (5)

where *Λ* is the de Broglie thermal wavelength, and *κ*_H_ is the Helfand compressibility. The final term is the coupling between the particles and the field, defined by

$$E^{pf}[\{r_{k}\},\rho ]\;=\;\int_{V}^{}dr\sum_{k}c_{k}Κ(r-r_{k})\rho (r).$$ (6)

A normalized Gaussian smoothing function is commonly adopted for the kernel function

$$Κ(r)\;=\;{\left(\frac{1}{\sigma^{2}\pi }\right)}^{3/2}\text{exp}\;(-r^{2}/\sigma^{2}).$$ (7)

Here, the scalar spread *σ* and coupling parameter *c*_*k*_ respectively describe the spatial extent and strength of interaction between the field and particle *k*. Assuming the solvent is always in a local equilibrium on a coarse-grained time scale, after a complicated derivation [62], the solvent-free potential between two particles *i* and *j* was obtained

$$E_{ij}\;=\;E^{p}(r_{ij})+U_{ij}(r_{ij}\text{)}\;=\;E^{p}(r_{ij}\text{)}-\frac{\tilde{c_{i}}\tilde{c_{j}}}{\kappa_{H}}{\left(\frac{1}{({\sigma_{i}}^{2}+{\sigma_{j}}^{2})\pi }\right)}^{\frac{3}{2}}e^{-\frac{1}{{\sigma_{i}}^{2}+{\sigma_{j}}^{2}}{r_{ij}}^{2}}.$$ (8)

Here, ${\tilde{c}}_{l}=s_{i}c_{i}$, and *s*_*i*_ is a scaling parameter. Besides ordinary particle-particle interactions, an additional attractive potential describing hydrophobic interactions is seen.

**Fig 1:**
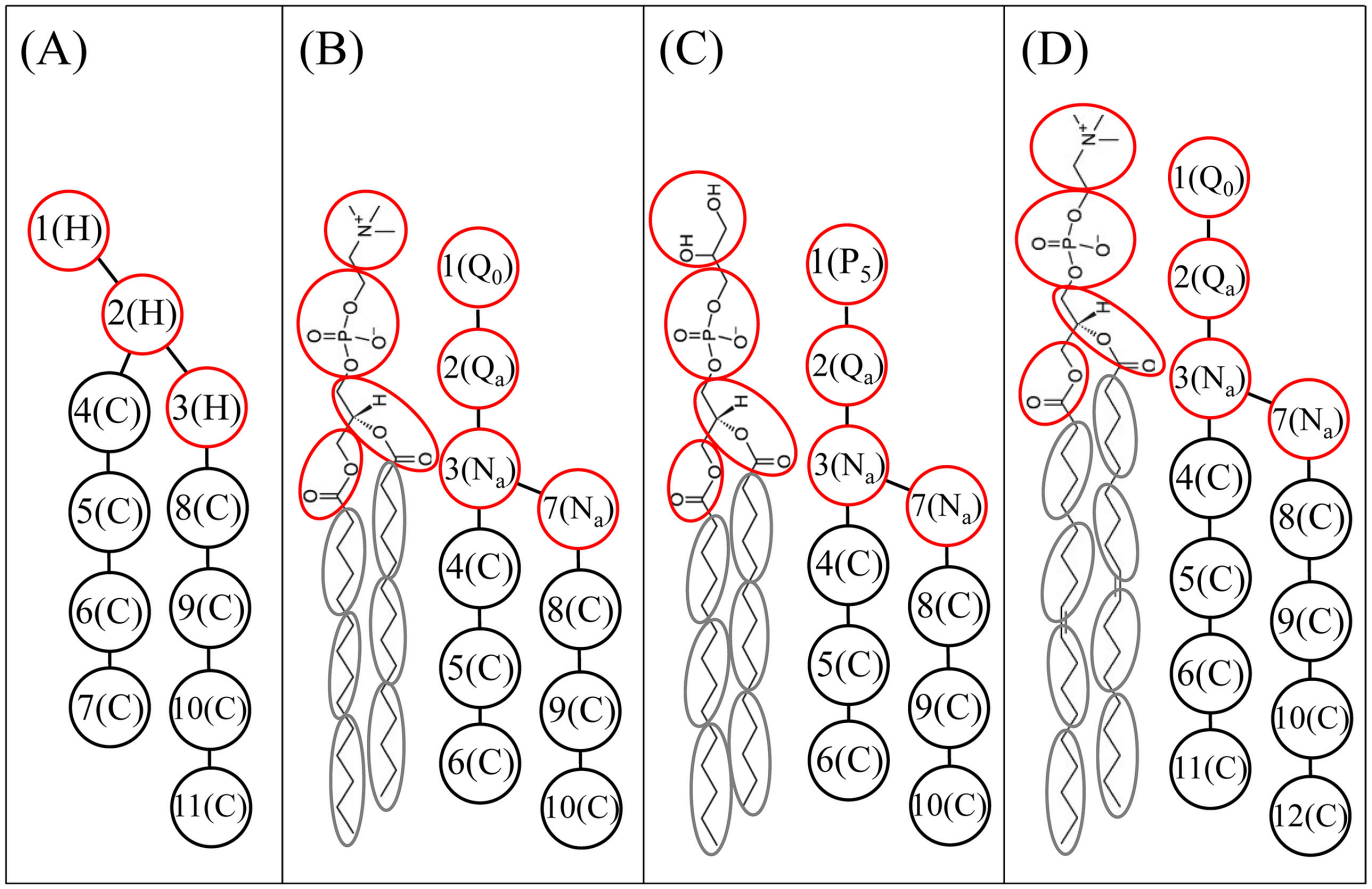
CG models of phospholipids. (A) λ-shaped DMPC. (B) h-shaped DMPC. (C) h-shaped DMPG. (D) h-shaped DOPC. Red and black circles refer to head groups and hydrophobic tails, respectively.

To describe the attractive potential, three variables must be parameterized for any type of CG bead, namely, the spreading width *σ*, the particle-field coupling strength *c*, and the scaling parameter *s*. Sevink and Fraaije constructed the parameters for a lipid bilayer membrane using a relatively simple CG lipid model wherein the CG lipid molecule took on a λ-shaped polymer H_3_(C_4_)_2_ composed of three head (H) particles and two hydrocarbon (C) tails of four particles each (see Fig 1A). They first explicitly obtained the field-particle coupling parameter *c* from existing DPD force parameters (*a*_ww_ = 25) for water-water interaction and the Helfand compressibility (*κ*_H_ = 4.6) via equation

$$c\;=\;0.095\Delta a+\kappa_{H},$$ (9)

where *a* = *a*_ww_ + Δ*a*. The DPD repulsive force parameter *a* for the H_3_(C_4_)_2_ lipid used in Ref. [31] is given as {*a*_WW_, *a*_WH_, *a*_WC_} = {25, 35, 75}, offering {*c*_H_, *c*_C_} = {5.55, 9.37}. The rescaled coupling parameter $\tilde{c}$ (or *s*) and spread *σ* were then obtained by applying the iterative Boltzmann inversion procedure to determine the effective interaction potential after removing a fraction of the particles. For Δ*a* = 0, the fitted $\tilde{c}$ in the concentration range of interest for liposome simulations was roughly equal to 4.6, the same as was found for the unscaled value for the reference system, suggesting that the previously determined additional scaling factor *s* was not necessary. The spread (*σ* = 0.4) was the same across the concentration range. In practice, for Δ*a* ≠ 0, Sevink and Fraaije suggested setting *s* = 1 and manually tuning *σ* to match the macroscopic membrane properties. They found membrane integrity to be most sensitive to *σ*_C_, the spread of the lipid tail bead, but membrane stiffness was sensitive to *σ*_H_, the spread of the lipid head bead. They showed that the determined membrane properties (area per lipid and bending rigidity) for the model with an explicit solvent are best reproduced for {*σ*_H_, *σ*_C_} = {1.11, 1.14}. However, when we tried to apply this method to a more complicated CG lipid model, such as a Martini-like h-shaped lipid (cf. Fig 1B–1D), it was difficult to tune the parameters to reproduce membrane properties. We therefore developed an alternative procedure for creating Im-DPD force parameters.

## Parameterization of Im-DPD potential for Martini-like CG lipids and peptides

**Fig 2:**
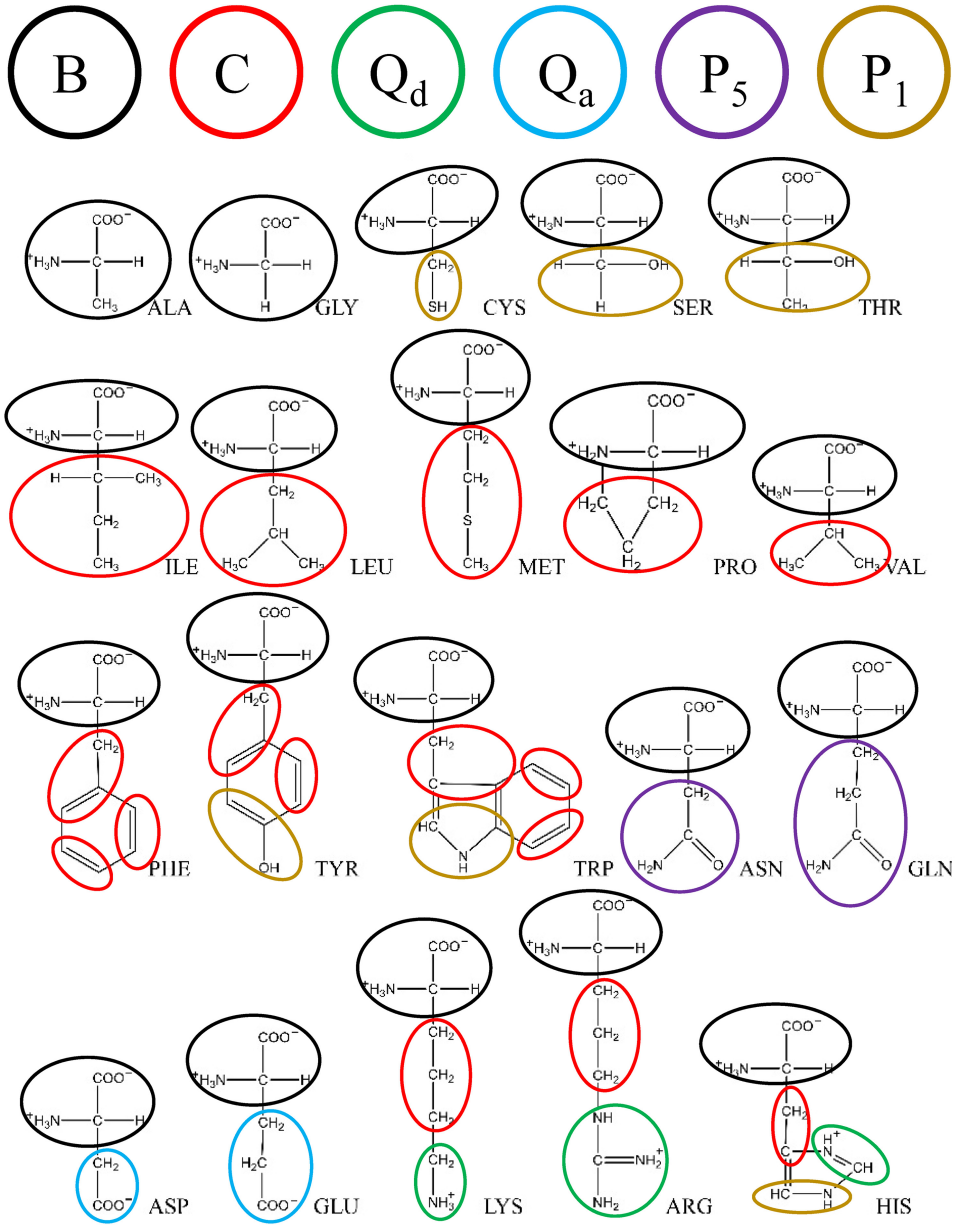
CG mappings for 20 amino acid residues.

We adopted a Martini-like CG model by mapping around four heavy atoms and their connected hydrogen atoms into one bead [47–49]. A lipid molecule was modeled as an h-shaped polymer connected by harmonic bonds and consisting of four hydrophilic head beads and two hydrophobic tails. The atomic representations and corresponding CG mappings of dimyristoyl phosphatidylcholine (DMPC), dimyristoyl phosphatidylglycerol (DMPG), and dioleoyl phosphocholine (DOPC) lipids are shown in Fig 1B–1D. The CG model for dipalmitoyl phosphatidylcholine (DPPC) lipid was constructed by adding one more bead to each of the two tails in the CG DMPC model. An amino acid residue was represented by one backbone bead and one or more side-chain beads, as shown in Fig 2. Based on functional group, as in the Martini model, the DPD beads were sorted into charged (Q), polar (P), nonpolar (N), and apolar (C) types [15]. Each type was further divided into subtypes based on hydrogen donor capacity (d), hydrogen acceptor capacity (a), or no hydrogen bond forming capacity (0). This CG model retains the essential chemical specificities of the molecules. Based on this four-to-one mapping scheme, we recently constructed a new set of Ex-DPD force fields [45], shown in Table 1. To reproduce the correct compressibility of water, a larger value, *a*_WW_ = 100, was assigned to beads of the same type. The repulsion parameters between beads of different types were obtained by matching to the Flory-Huggins *χ* parameter. Compared to the λ-shaped lipid model, this Martini-like DPD model allows for more types of CG beads. Therefore, an efficient and reliable approach is required for building an Im-DPD force field according to this CG mapping.

**Table 1:** DPD force parameters *a*_*ij*_ for Martini-like CG model.

*a*_*ij*_	W	Q_0_	Q_d_	Q_a_	N_a_	C	P_5_	P_1_	N_0_	N_da_
W	100	98	98	98	102	130	98	102	110	102
Q_0_	98	110	100	100	102	130	98	102	110	102
Q_d_	98	110	110	98	102	130	98	100	110	102
Q_a_	98	100	98	110	102	130	98	102	110	102
N_a_	102	102	102	102	100	110	102	102	110	102
C	130	130	130	130	110	100	130	110	110	110
P_5_	98	98	98	98	102	130	98	98	110	100
P_1_	102	102	100	102	102	110	98	100	102	102
N_0_	110	110	110	110	110	110	110	102	100	110
N_da_	102	102	102	102	102	110	100	102	110	100

We first optimized parameters *c* and *σ*, determining the coupling between the solvent field and the particles. For the special condition where only one particle is immersed in a homogenous and equilibrated solvent box, the density of the entire particle-field system is approximately equal to the density of the field *ρ*, which is 3/*r*_0_^3^ in this model. Thus, the force acting on the particle by a slice of solvent can be obtained analytically using Eqs (6) and (7) and has the form

$$f^{pf}(z)\;=\;-\frac{\partial E^{pf}}{\partial z}\;=\;-c\rho \frac{1}{\sigma \pi^{\frac{1}{2}}}[e^{\frac{{-\;(z_{1}-z)}^{2}}{\sigma^{2}}}-e^{\frac{{-\;(z_{2}-z)}^{2}}{\sigma^{2}}}].$$ (10)

**Fig 3:**
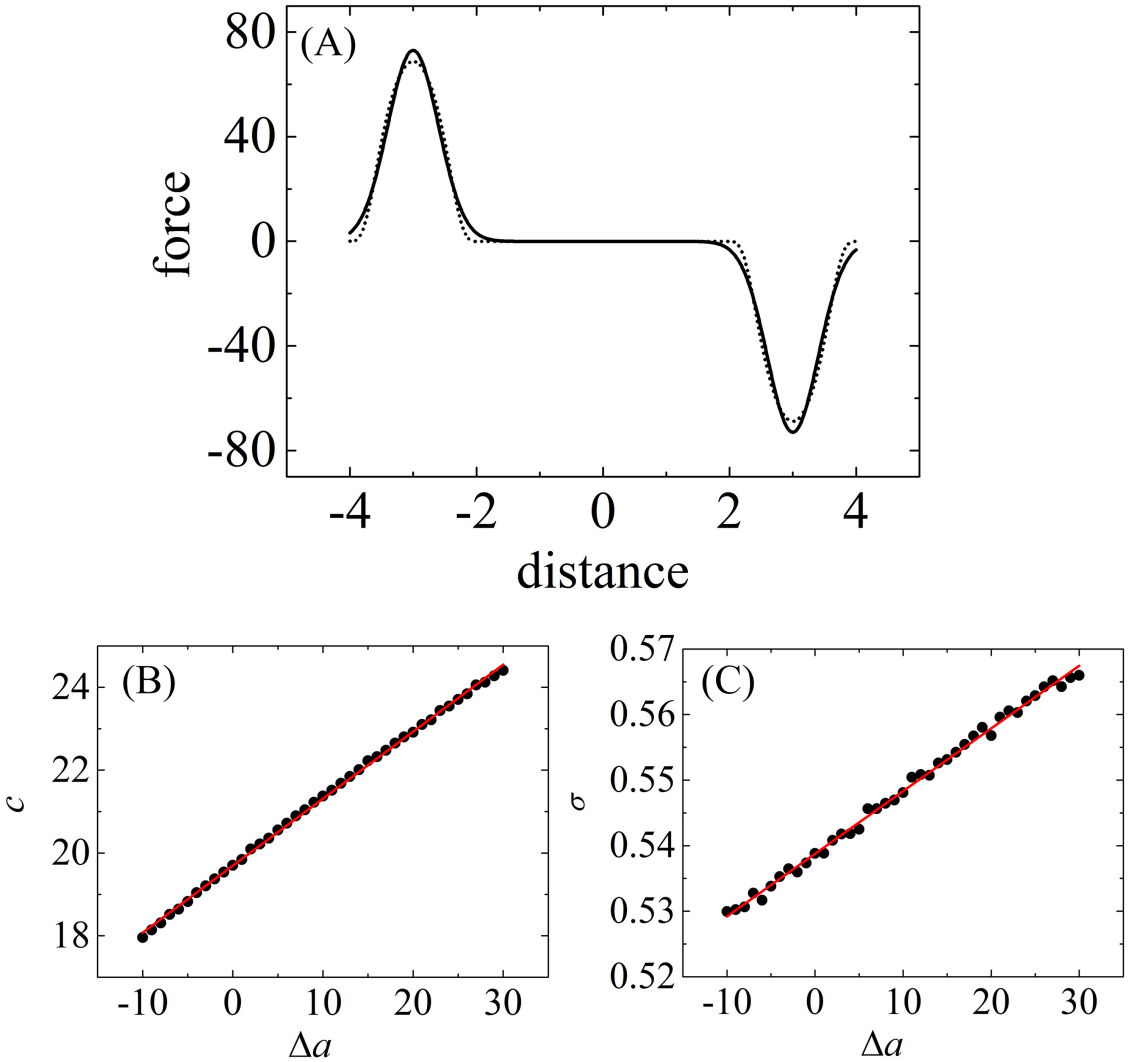
Im-DPD force parameters *c* and *σ* obtained from Ex-DPD simulations. (A) Gaussian curve fitting of the repulsive force between a C-type bead and a water slice as a function of distance between the particle and the center of mass of the water slice. Dash line is the simulation data. Solid line is the fitting result. (B) and (C) Linear fittings of *c* and *σ* as a function of the repulsive force difference.

Here, *z* is the distance between the particle and the center of the solvent slice, and *z*_2_-*z*_1_ is the thickness of the slice. This force can also be obtained using Ex-DPD simulation, as a sum of the forces between the particle and all the water beads of the slice, within a cut-off of 1.0 *r*_0_. To calculate the force, Ex-DPD simulations governed by Eqs (1)–(3) were performed in a constant volume and constant temperature (NVT) ensemble and periodic boundary conditions using the velocity Verlet algorithm. Here, the reduced temperature *T* was set to 1, corresponding to 298 K. The time step was set to 0.02*τ* ($\tau \;=\;r_{0}\sqrt{m_{0}/k_{B}T}$). The friction coefficient *γ*_*ij*_ remained consistent at 4.5 for any pair of beads. In this four-to-one CG model, *r*_0_ corresponds to 0.71 nm in physical units [28, 45]. Each simulation was performed for up to 2×10^5^ time steps. The final 10^5^ time steps, containing 1000 equally separated frames, were used for subsequent analysis. The force acting on the immersed bead was calculated by averaging all forces between this target bead and water beads inside the slice of thickness 4*r*_0_. The force on a bead, where Δ*a* = *a*_*i*w_—*a*_ww_ = 30, as a function of distance *z*, is shown in Fig 3A. This curve was well fitted by Eq (10) and gave rise to the pair {*c*, *σ*}. The forces for beads along a series of *a*_*i*w_ ranging from 90 to 130 were calculated to obtain the corresponding {*c*, *σ*}. As shown in Fig 3B and 3C, both increased almost linearly as a functions of Δ*a*. This is represented by equations

c=19.69+0.1619Δa, (11)

and

$$\sigma \;=\;0.5388+9.564\times 10^{-4}\Delta a.$$ (12)

From this fitting, we extracted the {*c*, *σ*} parameter set for all DPD bead types considered (Table 2). As in Eq (9), we set the Helfand compressibility to *κ*_H_ = 19.69 for this four-to-one CG model where *a*_ww_ = 100, almost four times the compressibility *κ*_H_ = 4.6 used in the Sevink and Fraaije model, where *a*_ww_ = 25. The value of *σ* was only half of that used by Sevink and Fraaije. We propose that *σ*, when in the range of 0.54–0.57 *r*_0_, is reasonable because in traditional DPD simulation, the dimensionless interaction cutoff *r*_0_ is only 1; *σ* cannot exceed this cutoff.

**Table 2:** Im-DPD force parameters *c*, *σ*, and *s* for Martini-like CG model.

	Q_0_	Q_d_	Q_a_	N_a_	C	P_5_	P_1_	N_0_	N_da_
*c*	19.37	19.37	19.37	20.02	24.56	19.37	20.02	21.31	20.02
*σ*	0.536	0.536	0.536	0.541	0.566	0.536	0.541	0.547	0.541
*s*	0.46	0.46	0.46	0.734	0.734	0.46	0.734	0.734	0.734

The scaling parameter *s* was introduced to yield the equilibrium density when particles have assembled to a condensed phase. This implies that *s* must be related to the system’s macroscopic properties. Therefore, we optimized *s* by first simulating the self-assembly process of DMPC vesicles then examining the structural and elastic properties of a planar bilayer membrane. A planar membrane was prepared by placing *N*_lipid_ = 1152 DMPC lipids into a grid spanning the *x*-*y* plane with their head groups facing outward and tail groups facing the membrane center. The three box dimensions (*L*_x_ = *L*_y_ = *L*_z_) were determined by *L*_x_ = (0.5*N*_lipid_*a*_prj_)^0.5^, where *a*_prj_ is the projected area per lipid at initial setup. To simulate vesicle assembly, a planar membrane inside a large box of 1.5*L*_x_×1.5*L*_y_×1.5*L*_z_ was prepared. A box this large can provide sufficient room for phospholipids to escape their periodic images, allowing the membrane to curl up along the edges with vesicle growth. The integration algorithm in Im-DPD is same as in Ex-DPD.

The implicit-solvent forces governing the motion of the DPD beads are described by

$${F_{ij}^{C}(r_{ij})}^{im}\;=\;F_{ij}^{C}(r_{ij})-\frac{2(s_{i}c_{i}){(s}_{j}c_{j})}{{\kappa_{H}\pi^{3/2}({\sigma_{i}}^{2}+{\sigma_{j}}^{2})}^{5/2}}(e^{\frac{-{r_{ij}}^{2}}{{\sigma_{i}}^{2}+{\sigma_{j}}^{2}}}){\hat{r}}_{ij},$$ (13)

$${F_{ij}^{D}(r_{ij})}^{im}\;=\;F_{ij}^{D}(r_{ij})+\gamma_{ij}^{im}{\left(e^{\frac{-{r_{ij}}^{2}}{{\sigma_{i}}^{2}+{\sigma_{j}}^{2}}}\right)}^{2}({\hat{r}}_{ij}∙v_{ij}){\hat{r}}_{ij},$$ (14)

and

$${F_{ij}^{R}(r_{ij})}^{im}\;=\;F_{ij}^{R}(r_{ij})-\sqrt{2\gamma_{ij}^{im}k_{B}T}(e^{\frac{-{r_{ij}}^{2}}{{\sigma_{i}}^{2}+{\sigma_{j}}^{2}}})\zeta_{ij}^{im}{\hat{r}}_{ij}.$$ (15)

The Im-DPD forces were pairwise as in Ex-DPD and conserved the overall momentum, in sharp contrast to typical Brownian Dynamics. Specifically, the cutoff *r*_c_^im^ was equal to 2.5 *r*_0_. A carefully tuned *γ*_*ij*_^*im*^ was assigned a value of 0.8 to maintain the temperature at 1.0 *k*_B_*T*. The random number $\zeta_{ij}^{im}$ differs from *ζ*_*ij*_ in usual DPD forces.

In a lipid molecule or peptide, the molecular skeleton was maintained via harmonic potentials, including 2-body bonding interactions and 3-body angular constraints. These are described by

$$E_{2}(r)\;=\;\frac{1}{2}K_{2}{\left(r-L_{0}\right)}^{2}$$ (16)

and

$$E_{3}(r)\;=\;K_{3}[1-\text{cos}\;\left(\theta -\theta_{0}\right)],$$ (17)

where *K*_2_ and *K*_3_ are the spring constants of the bond strength and angular stiffness, respectively, and *L*_0_ and *θ*_0_ are the equilibrium bond length and angular degree, respectively. The equilibrium CG bond lengths and angles and related force constants can be obtained by fitting the bond distributions into AAMD simulations [63]. Tables 3 and 4 list these constants for the typical lipid molecules and peptides.

**Table 3:** Equilibrium bonds, angles and corresponding force constants for DMPC, DMPG, DPPC, and DOPC lipids. The angle with the superscript a and b is for DPPC and DOPC lipid, respectively.

DMPC/DMPG			DPPC/DOPC		
bond	*L*_0_ (*r*_0_)	*K*_2_ (*k*_B_*T*/*r*_0_^2^)	bond	*L*_0_ (*r*_0_)	*K*_2_ (*k*_B_*T*/*r*_0_^2^)
1–2	0.47	512	1–2	0.47	512
2–3	0.47	512	2–3	0.47	512
3–7	0.31	512	3–7	0.31	512
3–4	0.59	512	3–4	0.59	512
4–5	0.59	512	4–5	0.59	512
5–6	0.59	512	5–6	0.59	512
7–8	0.59	512	7–8	0.59	512
8–9	0.59	512	8–9	0.59	512
9–10	0.59	512	9–10	0.59	512
			6–11	0.59	512
			10–12	0.59	512
angle	*θ*_0_ (degree)	*K*_3_ (*k*_B_*T*)	angle	*θ*_0_ (degree)	*K*_3_ (*k*_B_*T*)
2-3-4	180	6	2-3-4	180	6
2-3-7	120	6	2-3-7	120	6
3-4-5	180	6	3-4-5	180	6
4-5-6	180	6	4-5-6	180^a^, 120^b^	6
7-8-9	180	6	7-8-9	180	6
8-9-10	180	6	8-9-10	180^a^, 120^b^	6
			5-6-11	180	6
			9-10-12	180	6

**Table 4:** Equilibrium bonds, angles and corresponding force constants for amino acids: B stands for backbone bead and S represents side-chain bead.

amino acid					
bond	*L*_0_ (*r*_0_)	*K*_2_ (*k*_B_*T*/*r*_0_^2^)	bond	*L*_0_ (*r*_0_)	*K*_2_ (*k*_B_*T*/*r*_0_^2^)
*L*_BB_	0.40	512	His *L*_BS1_	0.34	512
Leu *L*_BS_	0.35	512	His *L*_S1S2_	0.20	512
Ile *L*_BS_	0.33	512	His *L*_S1S3_	0.20	512
Val *L*_BS_	0.24	512	His *L*_S2S3_	0.10	512
Pro *L*_BS_	0.31	512	Phe *L*_BS1_	0.33	512
Met *L*_BS_	0.47	512	Phe *L*_S1S2_	0.20	512
Cys *L*_BS_	0.33	512	Phe *L*_S1S3_	0.25	512
Ser *L*_BS_	0.22	512	Phe *L*_S2S3_	0.17	512
Thr *L*_BS_	0.24	512	Tyr *L*_BS1_	0.34	512
Asn *L*_BS_	0.34	512	Tyr *L*_S1S2_	0.20	512
Gln *L*_BS_	0.47	512	Tyr *L*_S1S3_	0.33	512
Asp *L*_BS_	0.34	512	Tyr *L*_S2S3_	0.19	512
Glu *L*_BS_	0.47	512	Trp *L*_BS1_	0.31	512
Arg *L*_BS_	0.34	512	Trp *L*_S1S2_	0.15	512
Arg *L*_SS_	0.35	512	Trp *L*_S1S3_	0.30	512
Lys *L*_BS_	0.35	512	Trp *L*_S2S4_	0.31	512
Lys *L*_SS_	0.28	512	Trp *L*_S3S4_	0.17	512
Cys-Cys *L*_SS_	0.46	512			
angle	*θ*_0_ (degree)	*K*_3_ (*k*_B_*T*)	angle	*θ*_0_ (degree)	*K*_3_ (*k*_B_*T*)
*θ*_BBB_ (β-sheet)	180	6			

To maintain the secondary structures of polypeptides, dissociable Morse potentials were applied to mimic hydrogen bond interactions between beads of a peptide skeleton that is helical or sheet-like. This is described by

$$E_{Morse}(r)\;=\;K_{M}{\left[1-e^{-\alpha (r-r_{M})}\right]}^{2}.$$ (18)

For a α-helical structure, MB_1-3_ Morse bonds were introduced between every two backbone beads separated by two normal harmonic bonds, and MB_1-5_ Morse bonds were introduced between beads separated by four bonds. The equilibrium distance *r*_M_ and depth of the potential well *K*_M_ were set to 0.60 *r*_0_ and 3.0 *k*_B_*T*, respectively, for bonds MB_1-3_ and 0.78 *r*_0_ and 6.0 *k*_B_*T*, respectively, for bonds MB_1-5_. In both cases, the width of the potential well *α* was set to 6.4 *r*_0_, and the cutoff for the Morse potential was 2.0 *r*_0_. To stabilize a β-sheet structure, a Morse potential with *K*_M_ = 6.0 *k*_B_*T*, *r*_M_ = 0.5 *r*_0_, and *α* = 6.4 *r*_0_ for every two hydrogen-bonded beads was applied. Simultaneously, a harmonic angle potential *θ*_0_ = 180° was applied for every two backbone beads separated by two bonds.

If both beads of a pair were charged, electrostatic interactions were calculated empirically [32] using

$$\frac{4\pi r_{\text{e}}U\left(r\right)}{\Gamma r_{\text{c}}}=\{\begin{array}{c} \frac{52}{35}-\frac{4}{5}{\left(\frac{rr_{\text{c}}}{r_{\text{e}}}\right)}^{2}+\frac{2}{5}{\left(\frac{rr_{\text{c}}}{r_{\text{e}}}\right)}^{4}-0.13587{\left(\frac{rr_{\text{c}}}{r_{\text{e}}}\right)}^{5.145}\;\;\;(r<r_{\text{e}}/r_{\text{c}})\\ \frac{r_{e}}{rr_{c}}-3.2100{\left(1-\frac{rr_{\text{c}}}{2r_{\text{e}}}\right)}^{6}\;\;\;\;\;\;\;\;\;\;\;\;(r_{\text{e}}/r_{\text{c}}<r<2r_{\text{e}}/r_{\text{c}})\\ \frac{r_{e}}{rr_{c}}\;\;\;\;\;\;\;\;\;\;\;\;\;\;\;\;\;\;\;\;\;\;\;\;\;\;\;\;\;\;\;\;\;\;\;(r>2r_{\text{e}}/r_{\text{c}}) \end{array}.$$ (19)

Here, *r*_c_ = *r*_0_ = 0.71 nm, representing the length unit. The electrostatic smearing radius *r*_e_ was set to 1.6 *r*_0_. At 298 K, the coupling constant $Г\;=\;\frac{e^{2}}{k_{B}T\epsilon_{0}\epsilon_{r}r_{c}}\;=\;65.78$ [32], where *ϵ*_0_ is the dielectric constant of a vacuum, and *ϵ*_r_ is the relative permittivity of water at room temperature. Because the water was implicit, the relative permittivity constant *ϵ*_r_ was only 15.0 [26] (in Ex-DPD, it was 78.3 for water).

**Fig 4:**
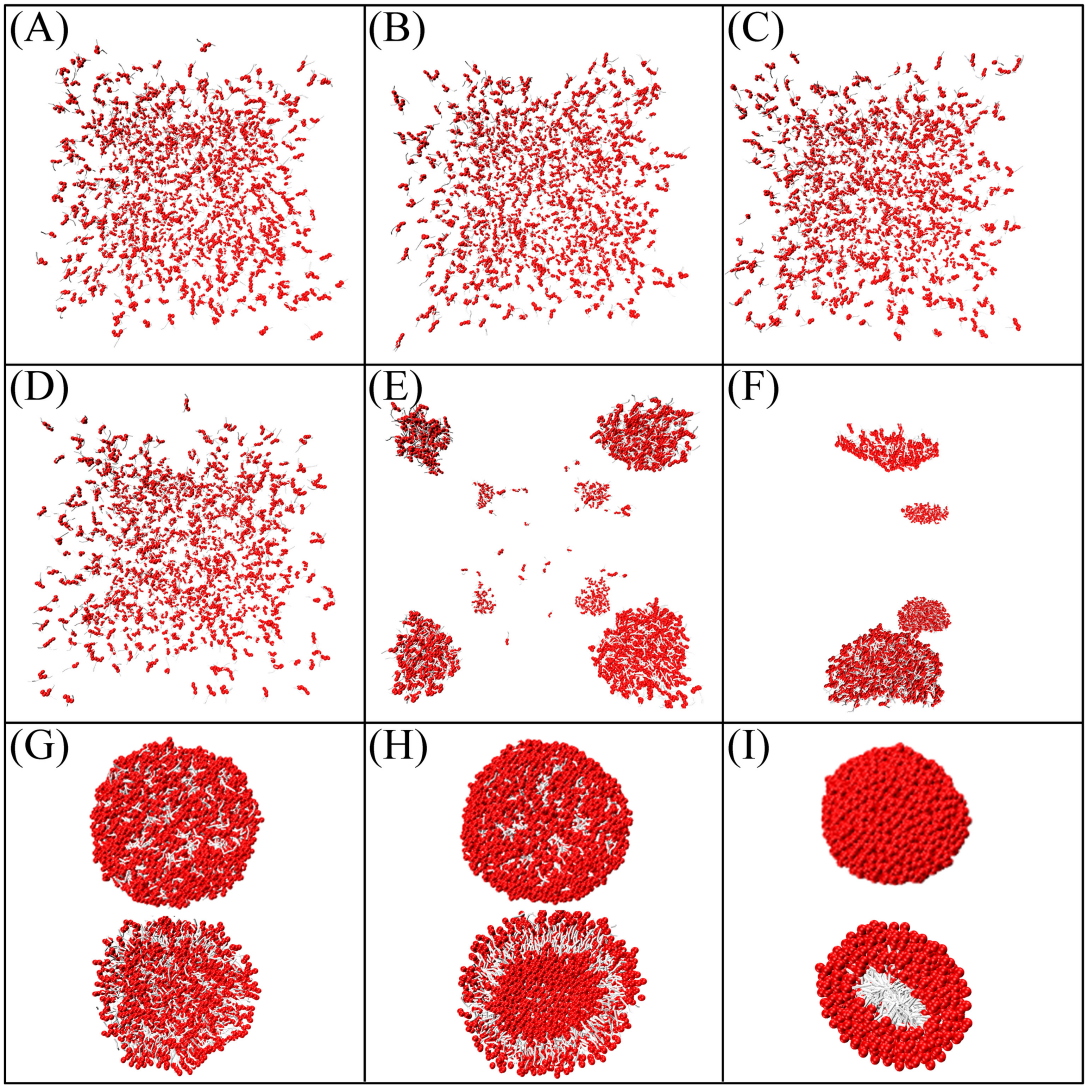
Snapshots of self-assemblies of DMPC lipids simulated by different scaling factor *s*. (A-I) *s* equals to 0.1–0.9. Cross-sectional views of half-cut micelles or vesicles are also given in (G-H).

**Fig 5:**
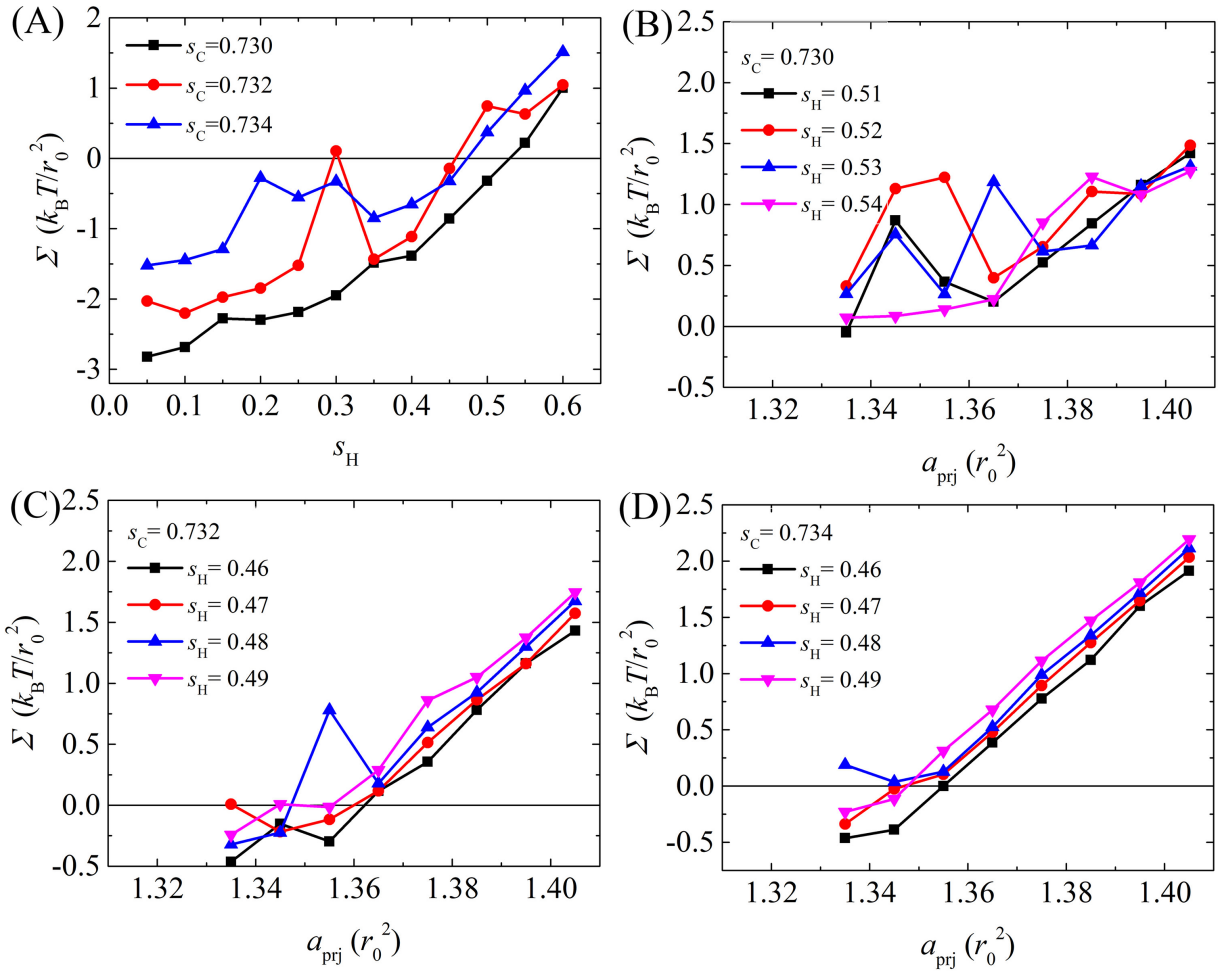
Membrane tension obtained by varying *s*_C_ and *s*_H_. (A) Membrane tension as a function of *s*_H_ for a bilayer with fixed *a*_prj_ = 1.355 *r*_0_^2^ and *s*_C_. (B-D) Membrane tension as a function of *a*_prj_ obtained at various {*s*_H_,*s*_C_} combination sets.

The parameter *s* was modulated so that the assembled structures of lipids could be examined. Initially, *s* was made equal for both head beads and tail beads. As illustrated in Fig 4, when *s* was small, the lipids were unable to assemble, but when it was large, tail particles densely packed inside the core of a hard spherical shape. Only when *s* ranged between 0.7 and 0.8 could the lipids self-assemble into a vesicle structure. However, the area per lipid of a tensionless planar bilayer *a*_prj_^0^ became 1.091 *r*_0_^2^ (detailed definitions of a tensionless bilayer and its area are forthcoming), much less than the 1.355 *r*_0_^2^ obtained in the Ex-DPD simulation [45]. To correctly reproduce the membrane area, *s* was finely tuned for the lipid head and tail separately. For simplicity, DPD beads were sorted only into hydrophilic (where *a*_*i*w_ is close to *a*_ww_) and hydrophobic (where *a*_*i*w_ is much larger than *a*_ww_) types while parameterizing *s*. After a series of simulations, a well-ordered vesicle and a tensionless bilayer with *a*_prj_^0^ close to 1.355 *r*_0_^2^ (Fig 5A) were found when {*s*_H_, *s*_C_} ranged between {0.46–0.54, 0.730–0.734}. To refine the parameters more precisely, the elastic response of a membrane to stress (i.e., the membrane tension as a function of area per lipid) was investigated. As shown in Fig 5B–5D, when *s*_C_ was set to 0.730 and 0.732 and *s*_H_ was in the pre-determined range, tension fluctuated unphysically upon stretching the membrane. In contrast, when *s*_C_ was set to 0.734, surface tension increased linearly with area per lipid. The area *a*_prj_^0^ was closest to 1.355 *r*_0_^2^ only when *s*_H_ = 0.46 among its reasonable values. The bending rigidity of a DMPC bilayer simulated via {*s*_H_, *s*_C_} = {0.46, 0.734} was 9 *k*_B_*T* ≈ 0.4×10^−19^ J, which is comparable to the Ex-DPD results (11 *k*_B_*T* ≈ 0.5×10^−19^ J [45]). These values for *s* were assigned to amino acid polypeptide beads based on their hydrophilicity and hydrophobicity (Table 2).

## Applications of Im-DPD

This new Im-DPD force field was applied to investigate the structural and elastic properties of three types of bilayer membranes, the free energy cost of moving a target molecule (a lipid molecule) or a molecular fragment (an amino acid analogue) into a bilayer membrane, and antimicrobial peptide (AMP)-induced membrane pore formation. The efficiency of this method was examined by simulating the self-assembly process of large vesicles.

### Bilayer properties

The bilayer’s tension as a response to stress was investigated first, then the membrane thicknesses, projected lipid headgroup areas, group density profiles, lipid order parameters, and bending rigidities of tensionless DMPC, DPPC, and DOPC bilayers were calculated.

To evaluate membrane tension as a function of the bilayer’s stretched area, planar membranes with fixed numbers of lipids but different initial projected areas per lipid *a*_prj_ (i.e., a different lateral area *L*_x_×*L*_y_) were simulated. In equilibrium, the tension *∑* of the bilayer was estimated via an integrated stress profile [31],

$$\Sigma \;=\;\int_{-\infty }^{+\infty }[P_{z}-0.5\times (P_{x}+P_{y})]dz,$$ (20)

where *P*_x_, *P*_y_, and *P*_z_ are the pressure components in three directions. A membrane with *∑* = 0 is said to be in the tensionless state. The corresponding projected area per lipid in the tensionless state is *a*_prj_^0^. The membrane thickness *L*_mem_ was obtained using the average distance between the choline groups in the upper and lower leaflets. The density distribution of relevant functional groups such as lipid head groups and lipid tail groups, perpendicular to the membrane (along the z-axis), was averaged along the x and y axes. The orientation order of the alkyl tails *S*_chain_ is defined in

$$S_{chain}\;=\;0.5\langle 3{cos}^{2}-1\rangle ,$$ (21)

where θ is the angle between the normal bilayer plane and the orientation of the vector along the hydrocarbon chain. The square bracket symbolizes an ensemble average.

**Fig 6:**
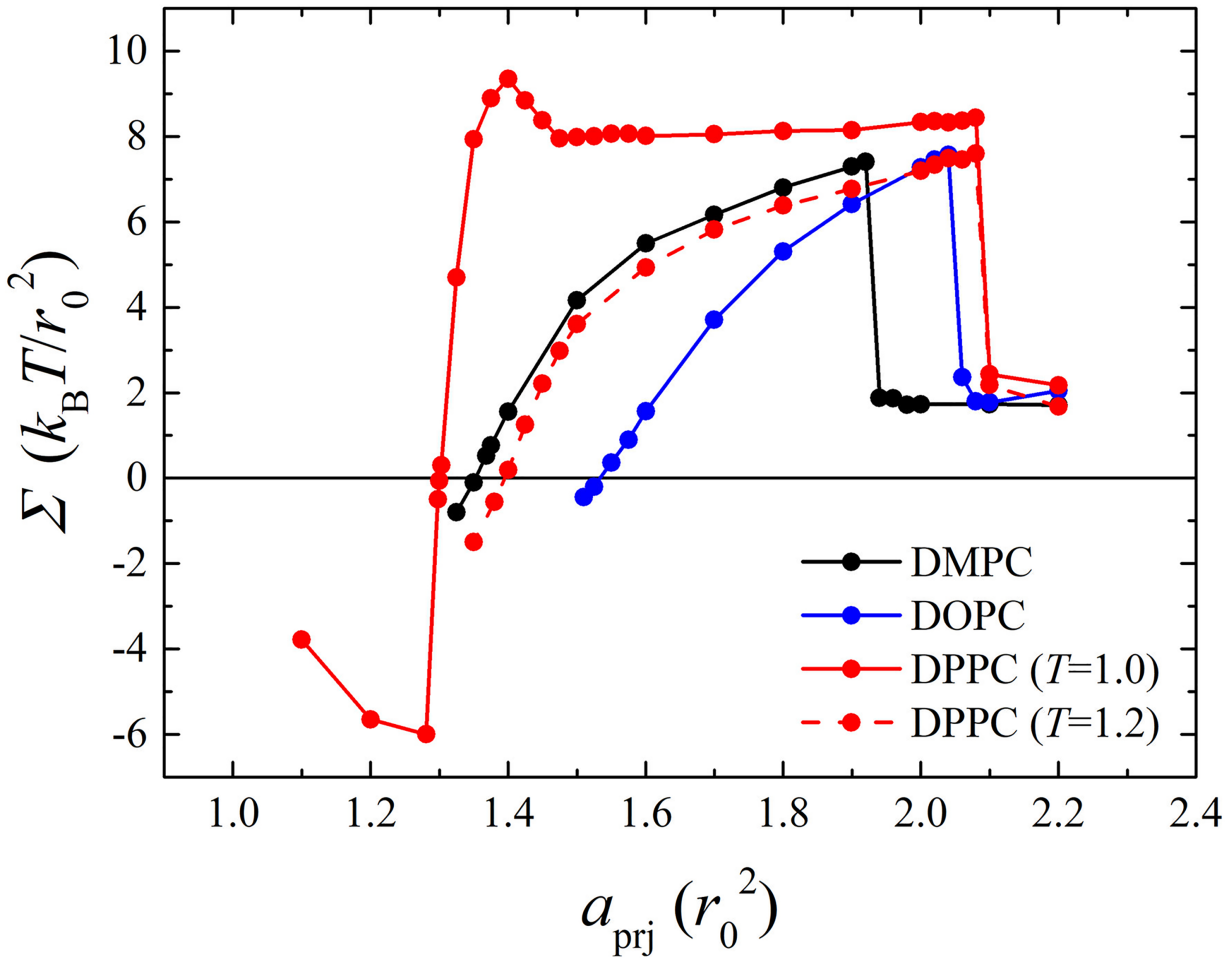
Membrane tension as a function of projected area per lipid *a*_prj_ for DMPC, DOPC, fluid phase DPPC and gel phase DPPC bilayers.

**Fig 7:**
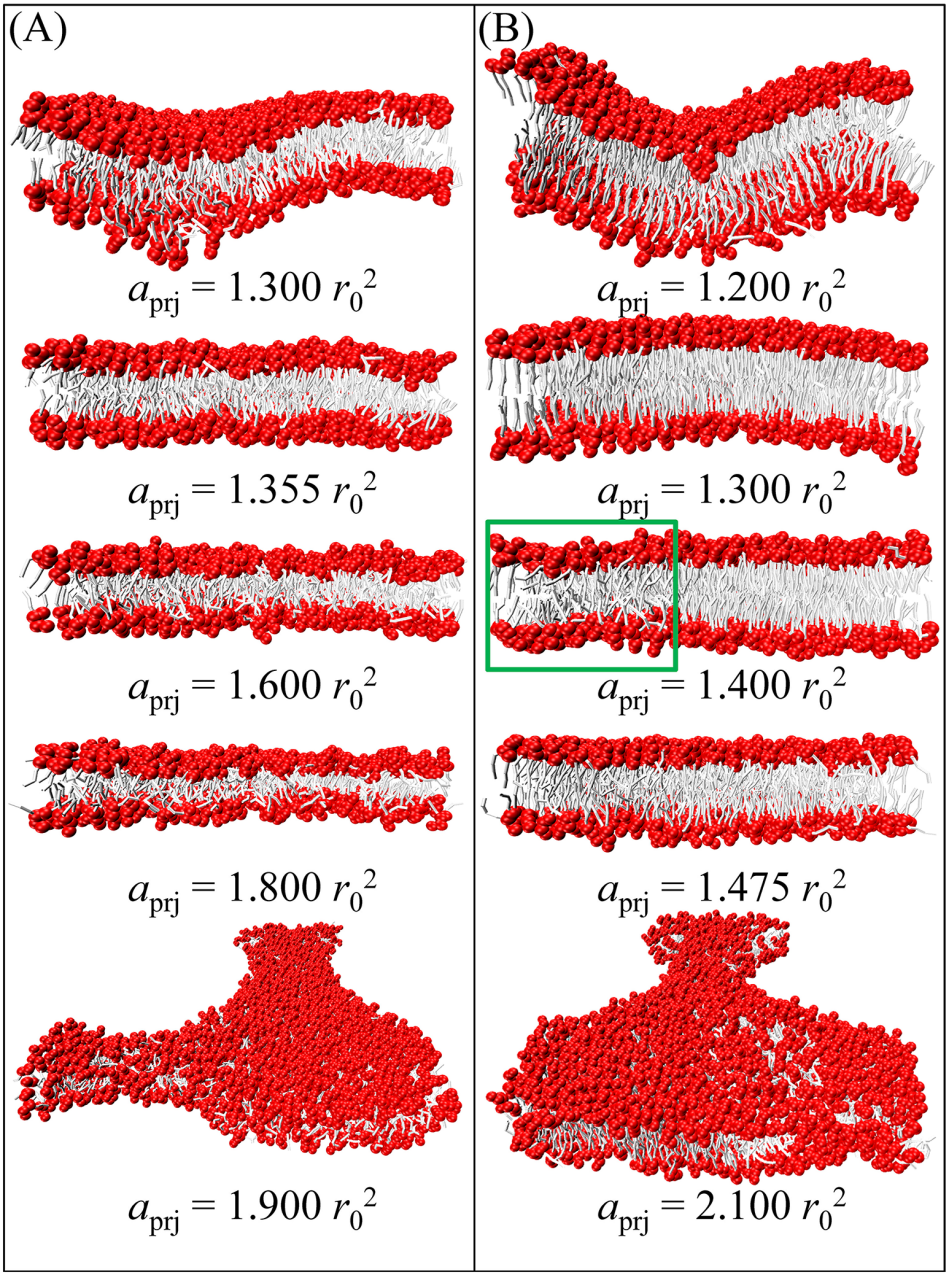
Snapshots of (A) fluid phase DMPC and (B) gel phase DPPC lipid bilayers at various *a*_prj_.

For DMPC, DOPC, DPPC (*T* = 1.0), and DPPC (*T* = 1.2) bilayers (each composed of 1152 phosphate lipids), series of Im-DPD simulations were performed in the NVT ensemble by varying initial *a*_prj_. The membrane tension as a function of *a*_prj_ is shown in Fig 6, and typical snapshots of the bilayers are presented in Fig 7. Simulations showed that at small *a*_prj_ (< *a*_prj_^0^), a membrane always undulates and tolerates negative tension. Increasing *a*_prj_ erased the wrinkles in the membrane, and it became stretched with positive tension. Tensionless projected areas per lipid *a*_prj_^0^ for these model bilayers, obtained from Fig 6, are listed in Table 5. Extreme stretching caused the membrane to rupture at a critical tension *∑*_rup_, also listed in Table 5. Once the membrane ruptured, its tension fell rapidly. Even though the values for tensionless *a*_prj_^0^ were different for DMPC, DOPC, and DPPC (*T* = 1.2) bilayers, their tension curves were similar and increased monotonically with membrane area before rupture, indicating they were in fluid phases. In contrast, the membrane tension for a DPPC bilayer simulated at *T* = 1.0 exhibited different elastic responses to stress. Near the tensionless state, tension increased quickly as the bilayer was stretched, as seen by a sharp slope in the curve. This indicated that the bilayer was in a gel phase. When the membrane area was stretched more than 7%, tension began to decrease. Once it was stretched close to 13%, tension increased again. Corresponding snapshots show that in the abnormal region, a local fluid phase (see Fig 7B) appeared in the bulk gel phase. The lipids in this local fluid phase were less compact and relatively easier to stretch than those in the gel phase; thus, the tension curve of the mixed fluid-gel phase membrane decreased when membrane area increased. The phase transition process quickly finished upon sufficient stretch, after which the DPPC bilayer behaved like a normal fluid-phase membrane. This direct membrane rupture upon stretching for fluid phase bilayers and phase transition under stress for gel phase bilayers were in good agreement with Ex-DPD results [45].

**Table 5:** Properties of Im-DPD lipid bilayers: Membrane thickness *L*_mem_ (nm), area per lipid *a*prj0 (nm^2^), order parameter of alkyl tails *S*_chain_, bending rigidity *κ* (10^−19^ J), and rupture tension *∑*_rup_ (mN/m). In the *κ* column, three values in sequence were from Im-DPD, Ex-DPD, and experiment.

lipid	*L*_mem_	*a*_prj_^0^	*S*_chain_	*κ*	*∑*_rup_
DMPC	4.15	0.68	0.67	0.4, 0.5[45], 0.56[54]	7
DOPC	4.38	0.77	0.38	0.4, 0.6[45], 0.4[55]	7
DPPC (*T* = 1.0)	5.10	0.66	0.91	8.0, 11.0[45], 10.0[53]	8
DPPC (*T* = 1.2)	4.80	0.70	0.64	0.4, 0.6[45], 1.0[53]	7

**Fig 8:**
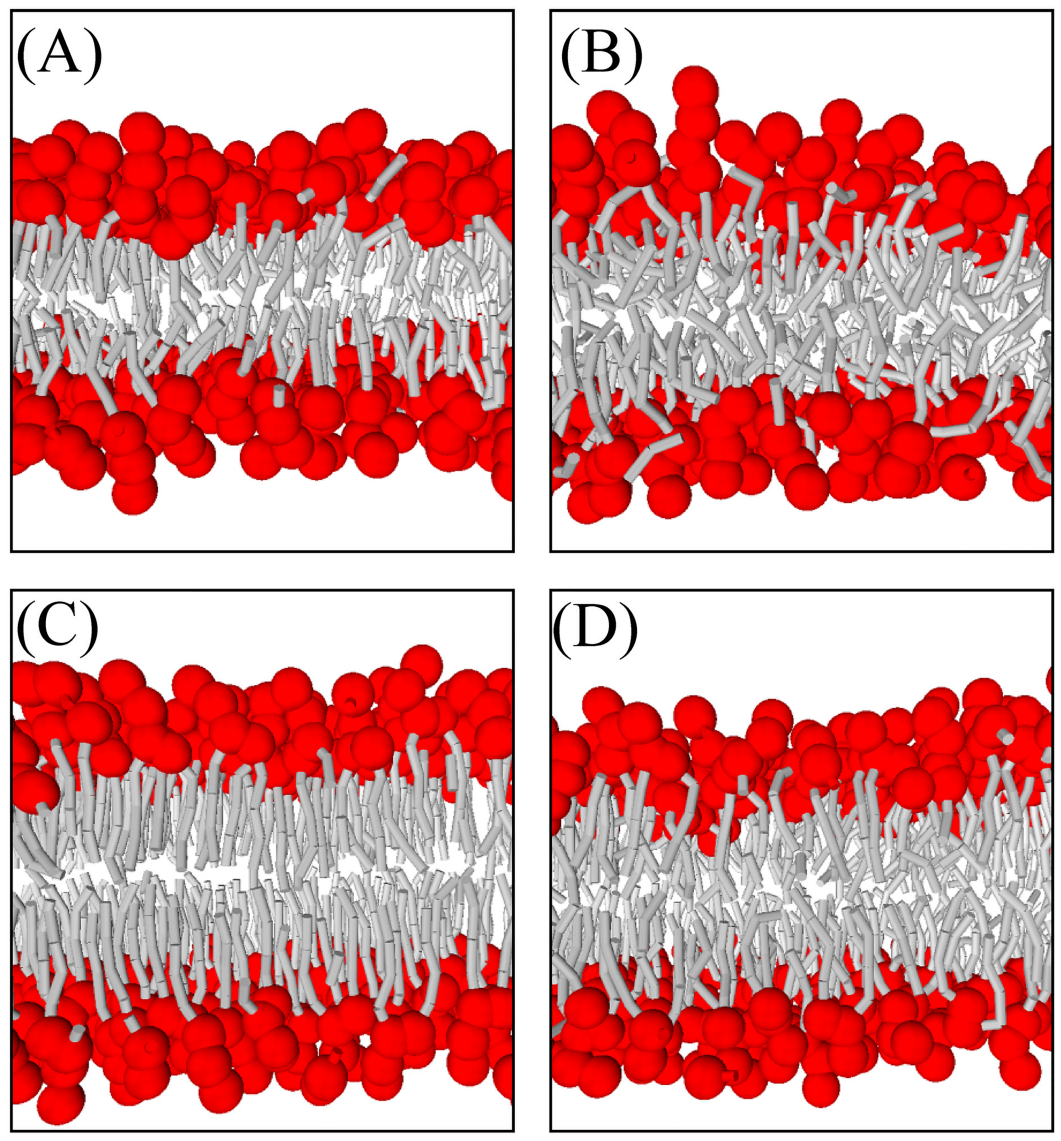
Snapshots of tensionless (A) DMPC, (B) DOPC, (C) gel phase DPPC, and (D) fluid phase DPPC bilayers.

**Fig 9:**
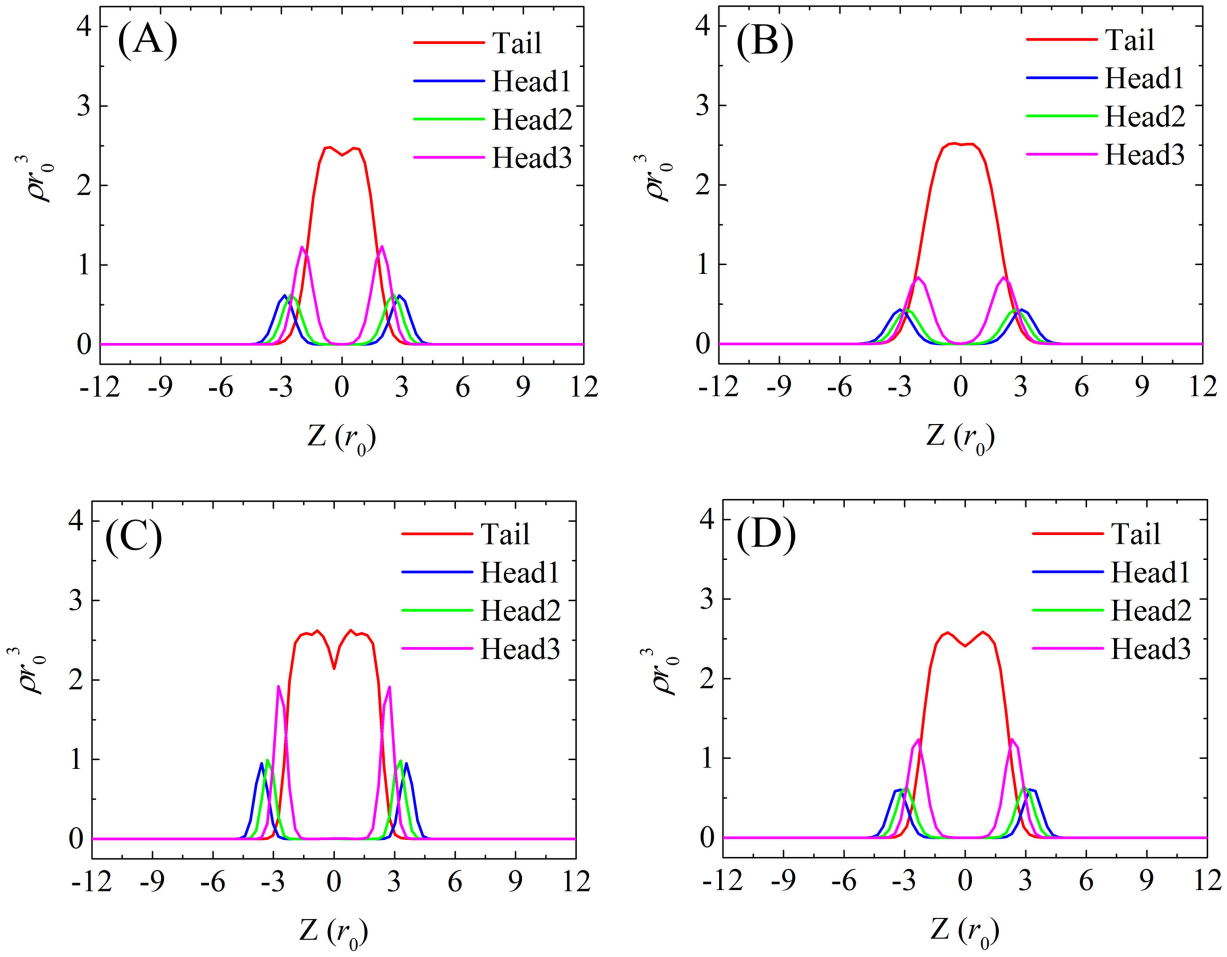
Density profiles of tensionless (A) DMPC, (B) DOPC, (C) gel phase DPPC, and (D) fluid phase DPPC bilayers along the direction of membrane normal.

More detailed snapshots and density distribution profiles of tensionless DMPC, DOPC, DPPC (*T* = 1.0), and DPPC (*T* = 1.2) bilayers are shown in Figs 8 and 9, respectively. As observed in Ex-DPD simulations [45], in the fluid phase, the lipid hydrophobic tails randomly spread out, and the terminal beads from opposite leaflets can touch each other. On the other hand, in the gel phase, the alkyl tails arranged like a hairbrush, and two leaflets greatly separated. The two distinguishable phases were further demonstrated by their order parameters of alkyl tails *S*_chain_ as shown in Table 5. The gel phase lipids arranged orderly and therefore had larger values for *S*_chain_ (as high as 0.9), but in the fluid phase, the tails beads were able to swing more freely and thus had slightly lower values for *S*_chain_ (less than 0.7).

A noticeable difference between Im-DPD and Ex-DPD models was the considerable vacuum (indicated by a minimum in the center of the tail bead distribution profile in Fig 9) arising at the centers of the membranes in Im-DPD simulations. In contrast, in Ex-DPD simulations [45], only shallow minima were observed for the gel phase DPPC (*T* = 1.0) bilayer. The tail bead distribution profiles for fluid phase DMPC, DOPC, and DPPC (*T* = 1.2) membranes were smooth. Based on these phenomena, the implicit-solvent bilayers were slightly thicker and had larger areas *a*_prj_^0^ than explicit-solvent bilayers, implying that implicit-solvent bilayers are less compact. This vacuum was frequently observed in atomistic membrane [50, 51, 64–66] and coarse-grained membrane [9, 10, 13, 26, 67]. The lack of vacuum in Ex-DPD is due to the short-range purely repulsive forces for every two interacting beads. It brings the lipids to assemble into compact structures to avoid the penetration of water. However, long-range L-J interaction in AAMD and other CGMD methods as well as a solvation term explicitly included in Im-DPD take into account the hydrophobic attractions, allowing less compact lipid packing. In this way, Im-DPD even compensates the imperfection of Ex-DPD.

Elasticity is another important property of lipid bilayers that influences cell growth, cell division, cell apoptosis, and so on. The fluctuation spectra method was used to identify the bending modulus of a membrane. In this method, the instantaneous positions of the choline groups were chosen, thus defining the two surfaces of the bilayer. The local height of the membrane *u*(*x*,*y*) is defined by

$$u(x,y)\;=\;\frac{1}{2}(Z_{1}(x,y)+Z_{2}(x,y)),$$ (22)

where *Z*_1_(*x*, *y*) and *Z*_2_(*x*, *y*) are the heights of the upper and lower surfaces, respectively. This bilayer was subsequently mapped to a coarser discrete grid with spacing *h* (*h* is typically assigned a value slightly larger than the membrane thickness). The discrete height *u*_*i*,*j*_ represents the average value of *u*(*x*, *y*) belonging to lattice (*i*, *j*). The Fourier expansion of the bilayer height is defined as

$$u(r)\;=\;\sum_{q}u(q)e^{iq∙r},$$ (23)

where ***q*** is the wave vector. The Fourier coefficients *u*(***q***) are given by

$$u(q)\;=\;\frac{h^{2}}{L_{x}L_{y}}\sum_{n}u(r)e^{-iq∙r}.$$ (24)

In the lattice model, ***r*** = *h****n***, ***n*** = *n*_*x*_***i*** + *n*_*y*_***j***, and $q\;=\;{\left(\frac{2\pi n_{x}}{L_{x}},\frac{2\pi n_{y}}{L_{y}}\right)}^{T}$, where $-\frac{L_{x}}{2h}\leq n_{x}<\frac{L_{x}}{2h}$, and $-\frac{L_{y}}{2h}\leq n_{y}<\frac{L_{y}}{2h}$. Radially averaging |*u*(***q***)|^2^ over *q* = |***q***| combined with time averaging over 1000 frames yields the fluctuation spectrum

$$S_{u}(q)\;=\;L_{x}L_{y}\langle {\left|u(q)\right|}^{2}\rangle .$$ (25)

This fluctuation spectrum is related to the membrane tension *Σ* and bending rigidity *κ* via

$$S_{u}(q)\;=\;\frac{k_{B}T}{\left(\kappa q^{4}+\Sigma q^{2}\right)}.$$ (26)

**Fig 10:**
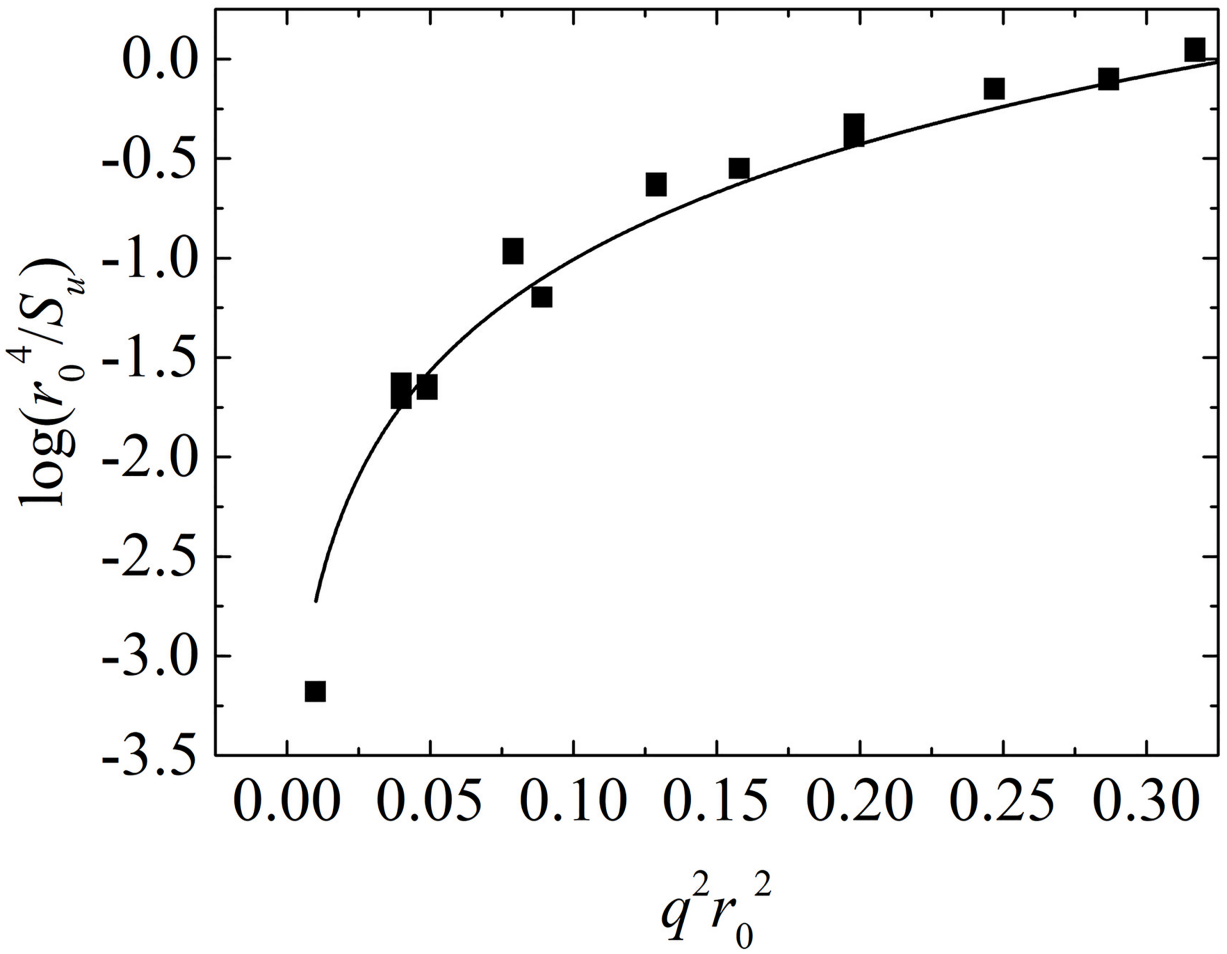
The logarithm of *r*04/*S*_*u*_ as a function of *q*^2^*r*02. Data were derived from a DMPC bilayer with *a*_prj_ = 1.355 *r*_0_^2^. Solid line is the fitting curve.

A larger membrane, where *L*_*x*_ = 2*L*_*y*_ = *L*_*z*_ ≈ 64*r*_0_, was prepared to guarantee sufficient membrane fluctuation. The membrane was then relaxed for up to 2×10^5^ time steps in the NVT ensemble. The final 1000 coordinates were collected for fluctuation analysis. A typical fluctuation spectrum for a tensionless DMPC bilayer is shown in Fig 10. By fitting Eq (26), *Σ* = 0.1 *k*_B_*T*/*r*_0_^2^ (close to 0), and *κ* = 9 *k*_B_*T* ≈ 0.4 × 10^−19^ J. The bending rigidities for DMPC, DOPC and DPPC (*T* = 1.0 and 1.2), obtained and shown in Table 5, were consistent with Ex-DPD results [45] and experimental data[53–55].

### Potential of mean force for flip-flop of a lipid molecule

A smaller bilayer containing 512 DMPC (or DOPC or DPPC) lipids was prepared to calculate the potential of mean force (PMF) for lipid flip-flop. By using the umbrella sampling method [68], a harmonic biasing potential was employed to restrain a lipid molecule (of the same type used for simulated membranes) at 61 continuous heights within the membrane, producing 61 serial simulation windows. The spacing between adjacent windows was set at 0.15 *r*_0_. The harmonic force with a constant of 110 *k*_B_*T* acted on the phosphate head bead of the lipid. After 10^5^ time steps of equilibration for each simulation, the weighted histogram analysis method [69] was utilized to produce PMF profiles from the subsequent 10^5^ time steps.

**Fig 11:**
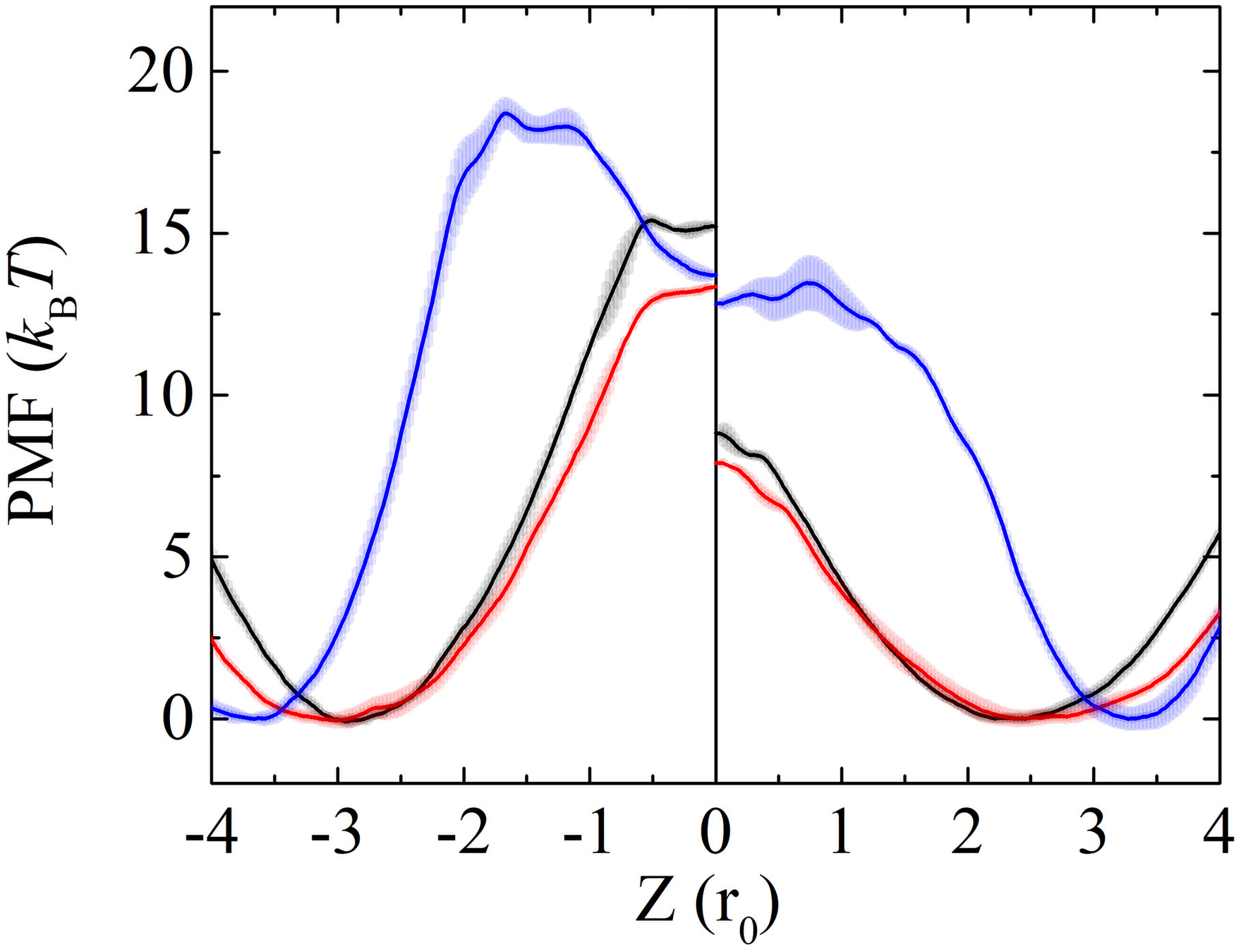
PMFs for DMPC (black), DOPC (red), and DPPC (blue) flip-flop in a bilayer obtained from Ex-DPD (right panel) and Im-DPD (left panel) simulations. Error bars indicate the standard error based on the asymmetry between the two leaflets of the bilayer.

The PMF profiles obtained from both Ex-DPD and Im-DPD are shown in Fig 11. Similar tendencies are seen: a free energy minimum appears near the head group where the confined lipid molecule is in equilibrium, a steep slope is seen as the head group moves into the bilayer center, and a flat plateau or a sharp peak is seen at the bilayer center. As one can see, the PMF profiles exhibited wide plateaus in the hydrophobic core region in Im-DPD and sharp peaks in Ex-DPD. Actually, both shapes were observed in the AAMD [70] and CG Martini simulations [51].

The energy differences for flip-flopping a DMPC, DOPC, and DPPC in Ex-DPD simulations are 16, 17, and 34 kJ/mol, respectively. These increase to 39, 34, and 47 kJ/mol, respectively, in Im-DPD simulations. Dozens of available free energy barriers from AAMD simulations [52, 66, 70], CG Martini simulations [51], as well as experimental excess chemical potentials [71] were summarized in Table 6. Besides DMPC, DOPC, and DPPC, the energy cost for DLPC flip-flop was also listed. Compared to DMPC lipid, a DLPC lipid has shorter tails with less two carbons in each tail, while they both have the same CG structures in either DPD or CG Martini method. The overall trend of the free energy barriers can be summarized as Ex-DPD<Im-DPD<AAMD≈Experiment<CGMD. It has been verified that, the PMF had a strong dependence on truncation radius [70], the treatment of electrostatic interactions [51], or even the force fields [70]. Taking the Berger DLPC lipid bilayer as an example, the increase of the cut-off from 0.9 nm to 1.4 nm brought about an increase of the energy barrier from 17 kJ/mol to 50 kJ/mol [70]. Another instance is the Martini membrane, wherein the normal water model estimated a free energy of 90 kJ/mol for DPPC flip-flop, which value increased to 160 kJ/mol in the polarized big multiple water model [51]. Even two AAMD lipids, e.g. Berger type and Charmm36 type, had a discrepancy as high as 40–50 kJ/mol [70]. Therefore, the differences between both DPD barriers and experiment references as well as AAMD estimates were within the realistic range. Both DPD underestimated the PMF barrier because they employed soft-core short-range interaction. The Im-DPD energy barrier was slightly greater than that of Ex-DPD because of the thicker Im-DPD membrane.

**Table 6:** Free energy barriers for lipids flip-flop. The symbol * denotes DOPG flip-flop in a pure DOPC membrane.

Δ*E* (kJ/mol)	experiment	AAMD	Ex-DPD	Im-DPD	CG Martini
DLPC	51[71]	17–65[70]	16	39	53–122[51]
DMPC	54[71]	39–95[70]			
DOPC	-	87[52], 94[66]	17	34	59–104*[51]
DPPC	69[71]	80[66]	34	47	90–150[51]

**Fig 12:**
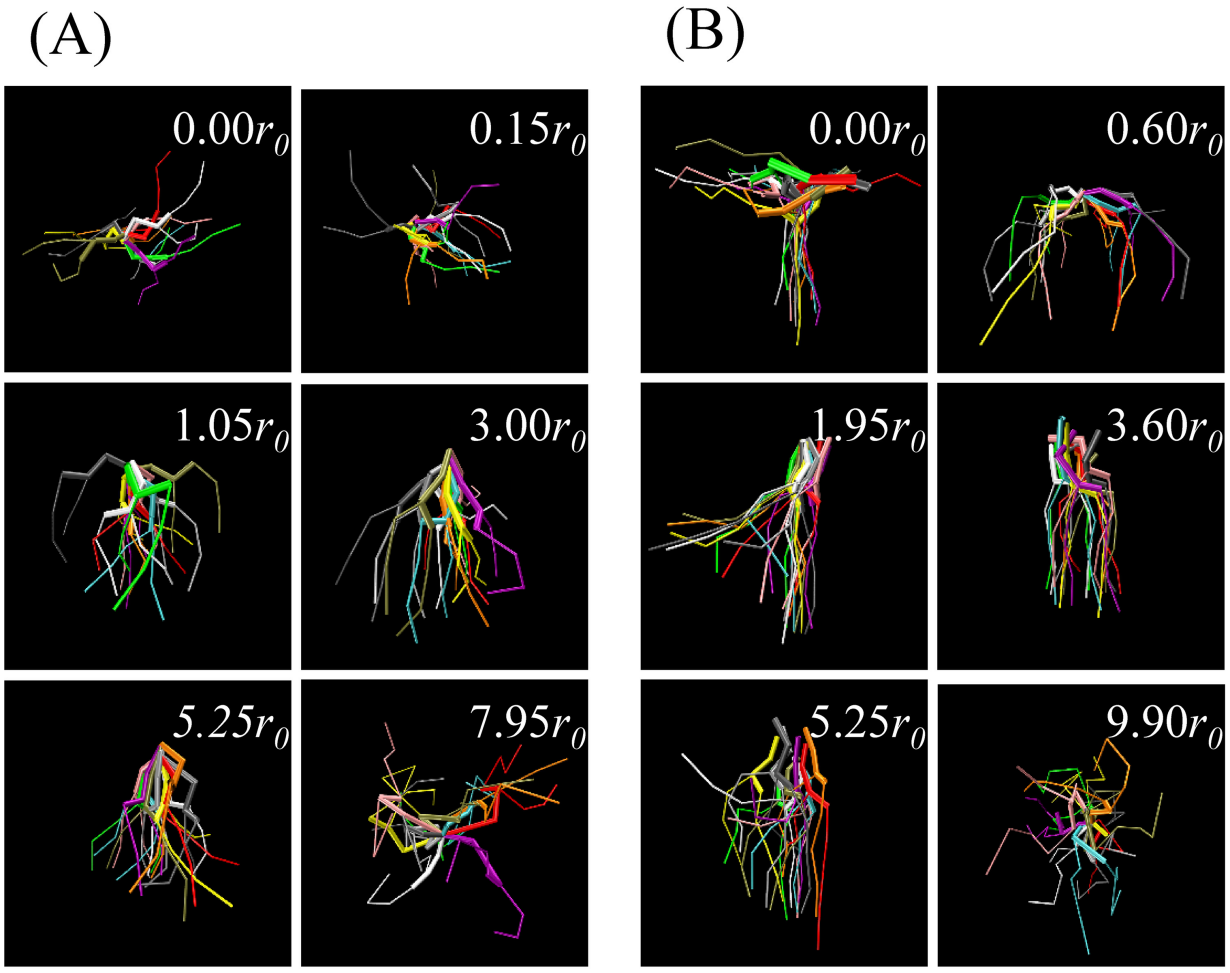
Snapshots of a restrained (A) DMPC and (B) DPPC lipid at different locations relative to the membrane center. At each location 11 independent frames in equilibrium states are presented. Different frames are represented by different colors. Thick bonds stand for head groups, while thin bonds stand for hydrophobic tails.

Specific snapshots for confined DMPC and DPPC lipids at various depths were taken from the simulations so that their conformations in fluid and gel phases could be compared. As shown in Fig 12, at membrane center (0.00 *r*_0_), the DMPC lipid became trapped in the vacuum region, and its tails were primarily oriented parallel to the membrane, but they could also interact with other lipids in both leaflets and have a variety of orientations. When pulled into equilibrium (3.00 *r*_0_), an elongated conformation was gradually adopted, resembling surrounding lipids. Forcing the lipid out of the membrane environment (7.95 *r*_0_) resulted in more random orientations. The DPPC lipid (Fig 12B) adopted a similar conformation distribution near the membrane surface (3.60 *r*_0_) and within the bulk implicit solvent (9.90 *r*_0_), while distinctions were seen near the membrane center (0.00 *r*_0_). One tail of the DPPC lipid was parallel and another tail was perpendicular to the normal membrane. In contrast, in Ex-DPD simulation, the two tails of DPPC splay to insert into two opposing leaflets separately. Once again, the larger vacuum in the bilayer center in Im-DPD allowed at least one tail to remain there.

### PMFs for CG amino acid beads across membranes

To verify the validity of the Im-DPD force field for polypeptides, the free energy cost for transferring side chain beads of amino acids across a DOPC bilayer membrane was investigated. For simplicity, 18 amino acid side chains (alanine and glycine lack side-chains) were sorted into 8 groups according to charge, hydrophilicity, and polarizability. They were the SER group (serine, threonine, and cysteine), VAL group (valine, leucine, isoleucine, methionine, and proline), ASN group (asparagine and glutamine), ASP group (aspartic acid and glutamic acid), LYS group (lysine and arginine), TRP group (tryptophan and tyrosine), PHE group (phenylalanine), and HIS group (histidine).

**Fig 13:**
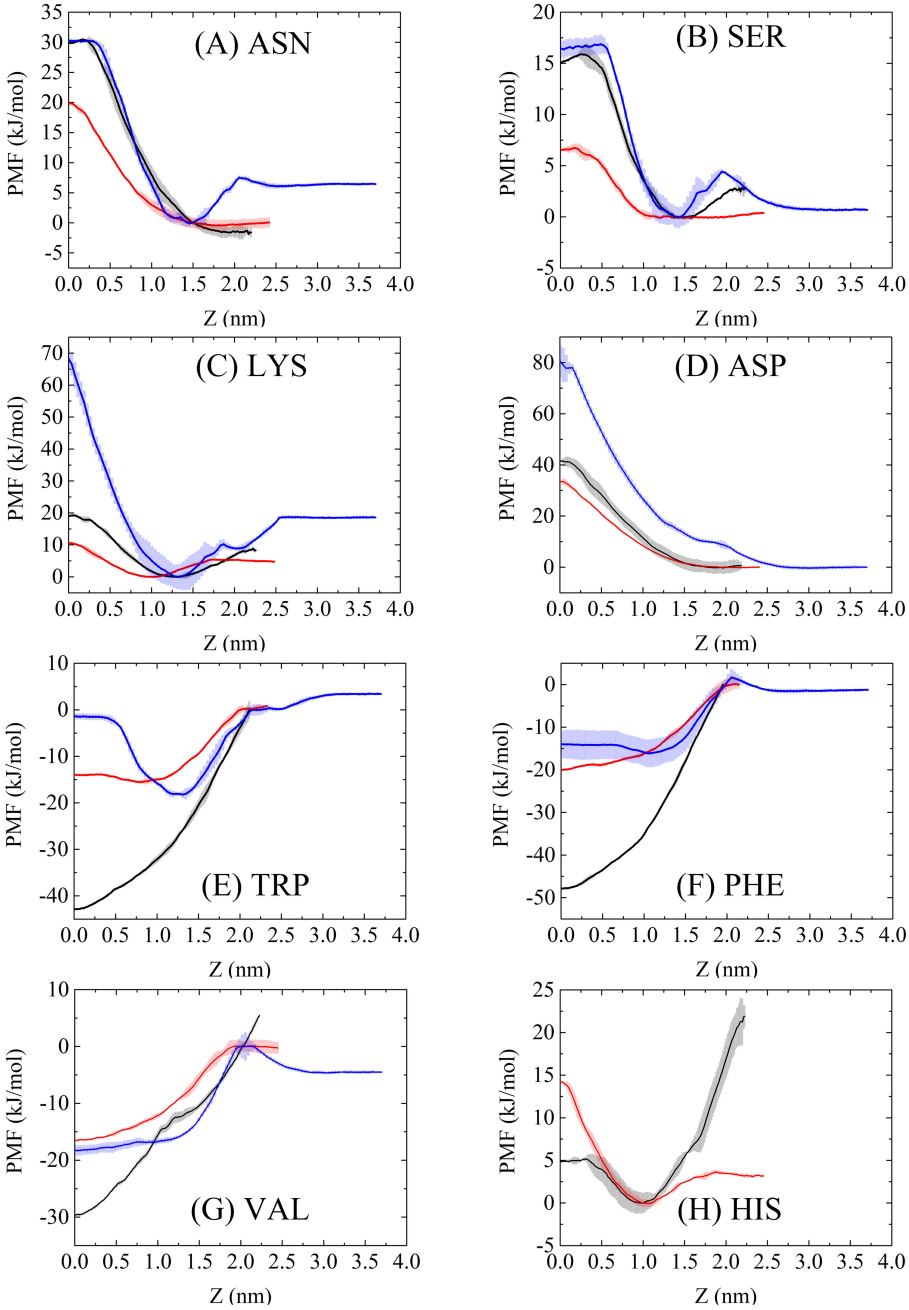
PMFs for moving amino acid side-chain analogues across a DOPC bilayer. Black, red, and blue lines in each figure refer to PMFs from Im-DPD, Ex-DPD, and AAMD [64, 65] simulations. Error bars indicate the standard error based on the asymmetry between the two leaflets of the bilayer.

Fig 13 presents the PMFs obtained from both Ex-DPD and Im-DPD simulations. The available PMF data from AAMD simulations are shown for comparison [64, 65]. To account for differences in the thickness of the membrane, the thickness of Ex-DPD and Im-DPD membrane was enlarged or shrunk to be consistent with AAMD membrane. Energy costs for transferring amino acid analogues from the bulk solvent to the membrane center are given in Table 7. The PMF profiles for polar uncharged ASN and SER groups (Fig 13A and 13B) showed energy peaks in the center of the bilayer and energy troughs in the bulk solvent, with a difference of less than 10 kJ/mol (e.g., 31.41–23.85 = 7.56 kJ/mol), providing a good agreement between atomistic and both DPD force fields. Using AAMD simulation, a free energy barrier appeared in the proximity of the lipid head group, implying the amphipathic nature of ASN and SER side-chains. However, in the coarse-grained method, ASN and SER side-chains were modeled simply as hydrophilic beads, suppressing any energy barrier.

**Table 7:** Energy barriers for transferring amino acid analogues from the bulk solvent to the membrane center obtained from Im-DPD, Ex-DPD, and AAMD [64, 65] simulations.

Δ*E* (kJ/mol)	Im-DPD	Ex-DPD	AAMD[64, 65]
ASN	31.41	20.15	23.85
SER	12.62	6.63	15.79
LYS	12.25	5.21	49.63
ASP	41.61	33.46	80.59
TRP	-39.19	-13.93	-4.79
PHE	-47.88	-19.70	-12.78
VAL	-29.25	-16.54	-13.75
HIS	-12.18	10.72	

The PMFs for charged residues (LYS and ASP) showed relatively large differences between AAMD and DPD simulations (Fig 13C and 13D). The free-energy penalties for burying the residues inside the membrane by the atomistic force field were much larger than those found using either Ex-DPD or Im-DPD force field. The CG bead model carrying a single charge underestimated the hydrophilicity of a charged residue.

As shown in Fig 13E and 13F, the aromatic residues of TRP and PHE entered into the hydrophobic membrane center more easily in DPD simulations. In the DPD model, TRP and PHE side-chains were represent by 2 to 3 C-type beads, which are prone to be compatible with lipid tails. However, a barrier in the center of membrane in the AAMD simulation indicated the difficulty in maintaining a large ring residue even though it is hydrophobic. The CG model does not reproduce this characteristic.

In the process of transferring a VAL into the bilayer center, all three models were thermodynamically admissible (Fig 13G). Although VAL has a PMF very similar to those of TRP and PHE, because all are primarily composed of C-type beads, it differs in that it is smaller, comprising only one CG bead. As a result, VAL had fewer PMF discrepancies between the DPD and AA models.

In the PMF profile of HIS, atomistic references were unavailable. In DPD representations, its side-chain has a ring structure linked by P_1_-, Q_d_-, and C-type particles, making it is amphiphilic. As a result, the PMF curve had a minimum at the hydrophilic-hydrophobic interface of the bilayer.

Overall, the free energy of transferring an amino acid side-chain across a lipid bilayer obtained using the Im-DPD model was satisfactory, reproducing the Ex-DPD results well. However, a large discrepancy as high as 25 kJ/mol was obtained for the aromatic ring residue, e.g. TRP, PHE, and HIS, while 6 to 13 kJ/mol was for the rest, in terms of the difference between two DPD methods. We speculate this was of the same reason as between Martini and Dry Martini [26]. No matter in Martini or DPD force field, the aromatic beads partly overlap. Thereupon, the implicit description for water was overestimated, unwantedly improving the hydrophilicity of aromatic rings, also bringing larger energy costs. As mentioned already, the PMF data had strong dependence on the force fields and simulation conditions. For most amino acids, the barrier differences obtained from Ex/Im DPD simulations were within the realistic range compared to AAMD estimations. Nevertheless, room for improvement remains. For example, a more precise scaling factor *s* could be introduced for specific CG beads.

### Antimicrobial peptide-induced membrane pore formation

Antimicrobial peptides are small and electropositive peptides with antimicrobial activities against many pathogenic microorganisms such as fungi, bacteria, etc. DPD simulation at the mesoscopic level provide a better understanding of the relationship between AMP structures and their actions. For example, we recently employed an Ex-DPD method to simulate the interactions between membranes and a couple AMPs with various secondary structures [49]. In this work, we investigated membrane pore formations induced by magainin and melittin as a practical application of Im-DPD.

**Fig 14:**
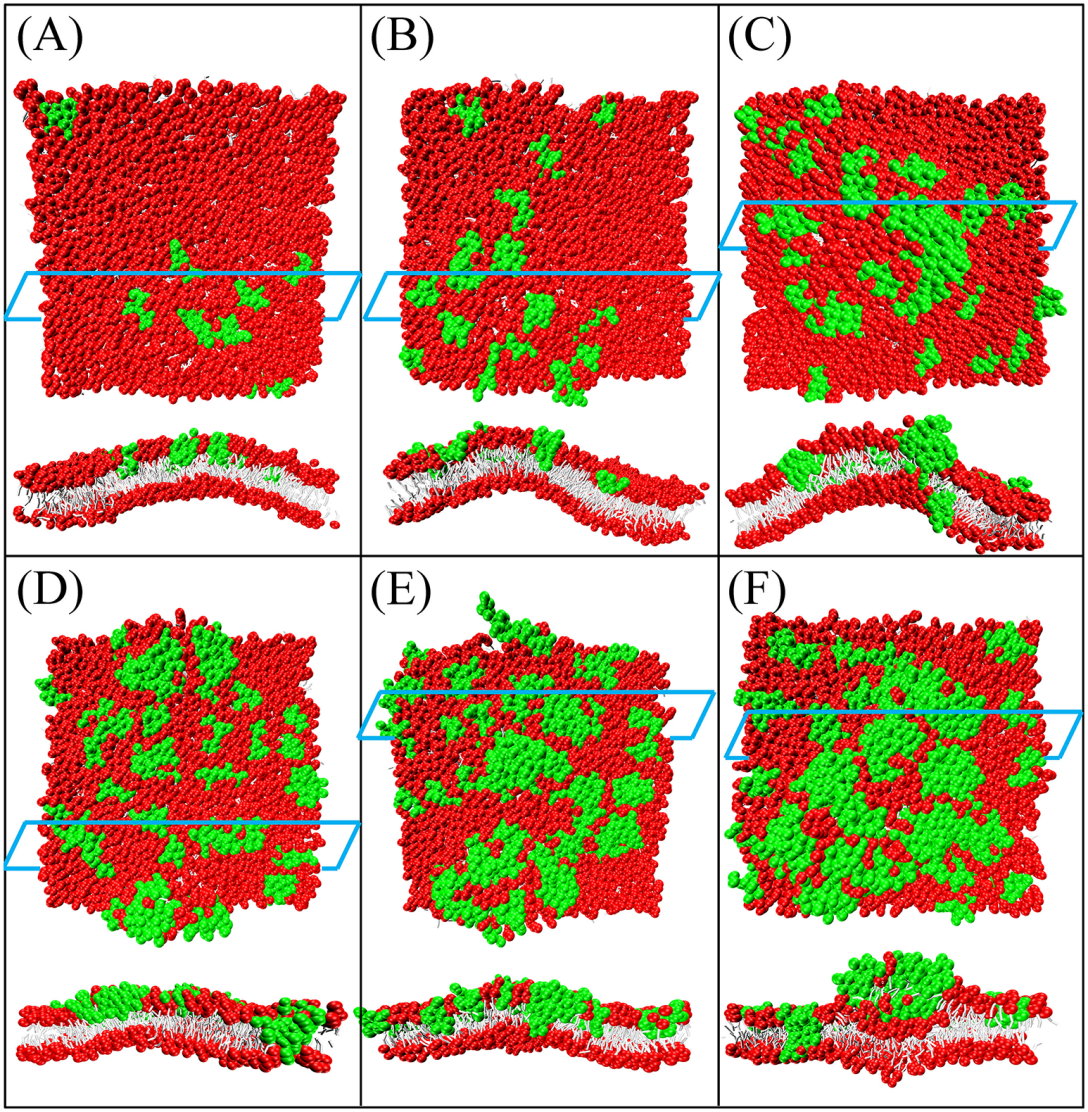
Top and cross-sectional views of magainin-induced membrane deformation or perforation at P/L molar ratio of (A) 0.5%, (B) 1%, (C) 2%, (D) 3%, (E) 4%, and (F) 5%. Snapshots were obtained at simulation time of 2×10^5^ time steps. Magainin peptides are in green color.

**Fig 15:**
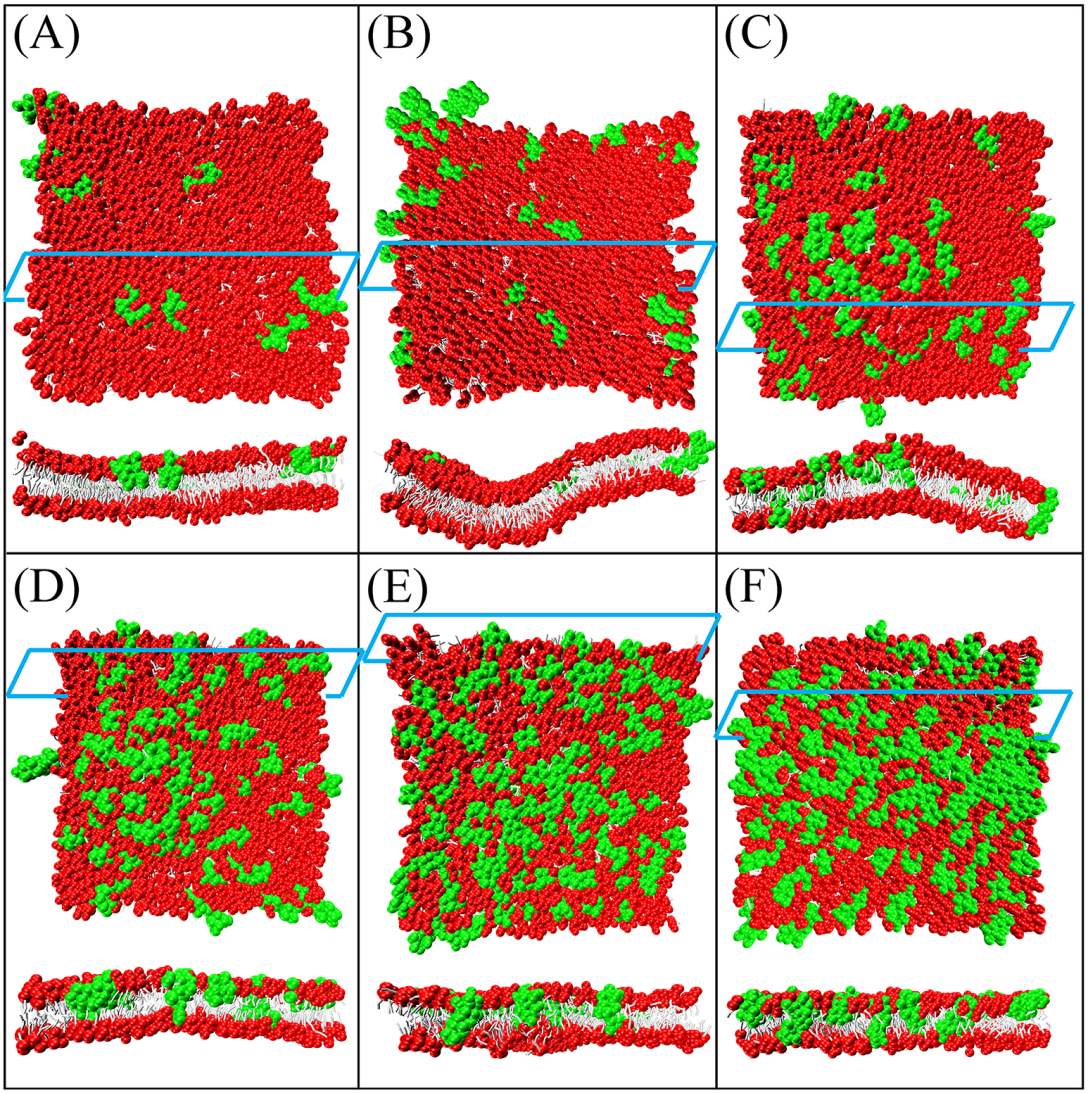
Top and cross-sectional views of melittin-induced membrane deformation or perforation at P/L molar ratio of (A) 0.5%, (B) 1%, (C) 2%, (D) 3%, (E) 4%, and (F) 5%.

Similar to the work of Ref. [49], the secondary structures of magainin and melittin were maintained as helixes to resemble their conformations upon binding to the membrane surface. A planar bilayer composed of 1682 zwitterionic DMPC and anionic DMPG lipids (DMPC:DMPG = 7:3) was used to mimic a bacterial membrane. A series of simulations were performed at peptide/lipid (P/L) molar ratios of 0.5%, 1%, 2%, 3%, 4%, and 5%. Counterions were loaded to neutralize the system. Results were consistent with those found using Ex-DPD simulation [49]. As shown in Fig 14, when the magainin concentration was low, the absorbed AMPs induced membrane buckling to a small degree. When more peptides were bound onto the membrane surface, the buckling was considerable, and the lipids were strongly disordered. To relax the peptide-induced compression (or tension), one or two magainin peptides were inserted into the membrane to form a small pore. Additional peptides and lipid head groups were added to enlarge the pore. Melittin peptides also induced pore formation in membranes with sufficient absorptions (Fig 15). Because the charge distribution along the helix was more discrete, magainin induced larger pores composed of 5 to 6 peptides. On the other hand, the charge on melittin was located primarily near the C-terminus, inducing the formation of smaller pores (each of 2–4 peptides). The shape and size of the peptide-induced pore and the critical peptide concentration necessary to induce pore formation in Im-DPD were good reproductions of those found using Ex-DPD [49] and AAMD [72, 73], as listed in Table 8.

**Table 8:** Membrane pores induced by magainin and melittin: The threshold pore forming peptide concentration C_pep_, the pore model, the number of peptides inside a pore N_pep_, and the inner diameter D_pore_. DT denotes the disordered toroidal model [74], B denotes the barrel-stave model [75], and C denotes the carpet model [76].

		Pore Properties	Im-DPD	Ex-DPD [49]	AAMD [72, 73]
Magainin	C_pep_ (%)		~2	~1	~3
	pore model		DT or B	DT or C	DT
	N_pep_		5–6	5–6	~4
	D_pore_ (nm)		2.5–3	1.8–3.6	~2
Melittin	C_pep_ (%)		~1	~1	~2
	pore model		DT or B	DT	DT
	N_pep_		2–4	2–4	~3
	D_pore_ (nm)		1.5–2	< 1.8	2.8–3.5

### Efficiency of Im-DPD

**Fig 16:**
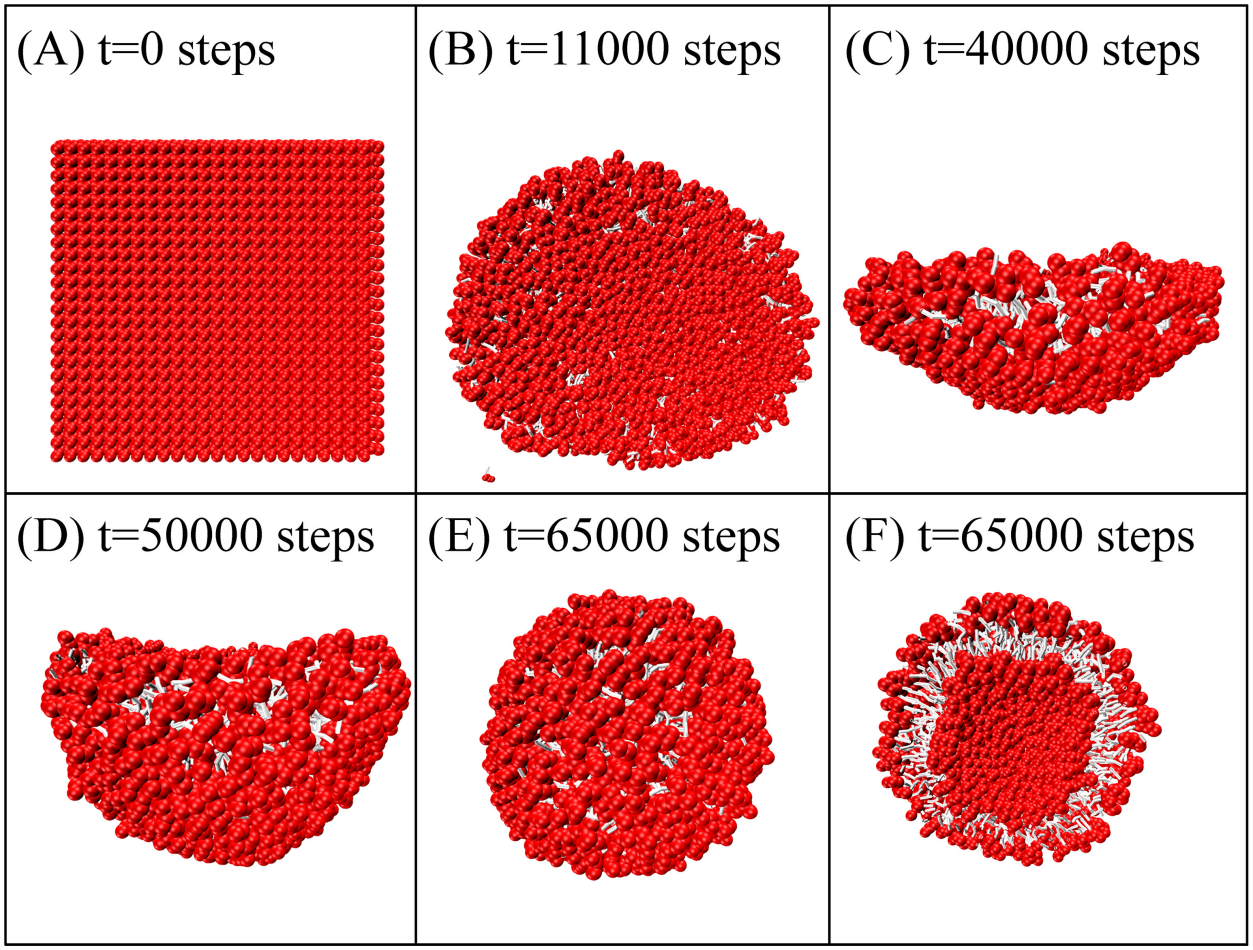
Time evolution of the formation of a vesicle composed of 1152 DMPC lipids.

**Fig 17:**
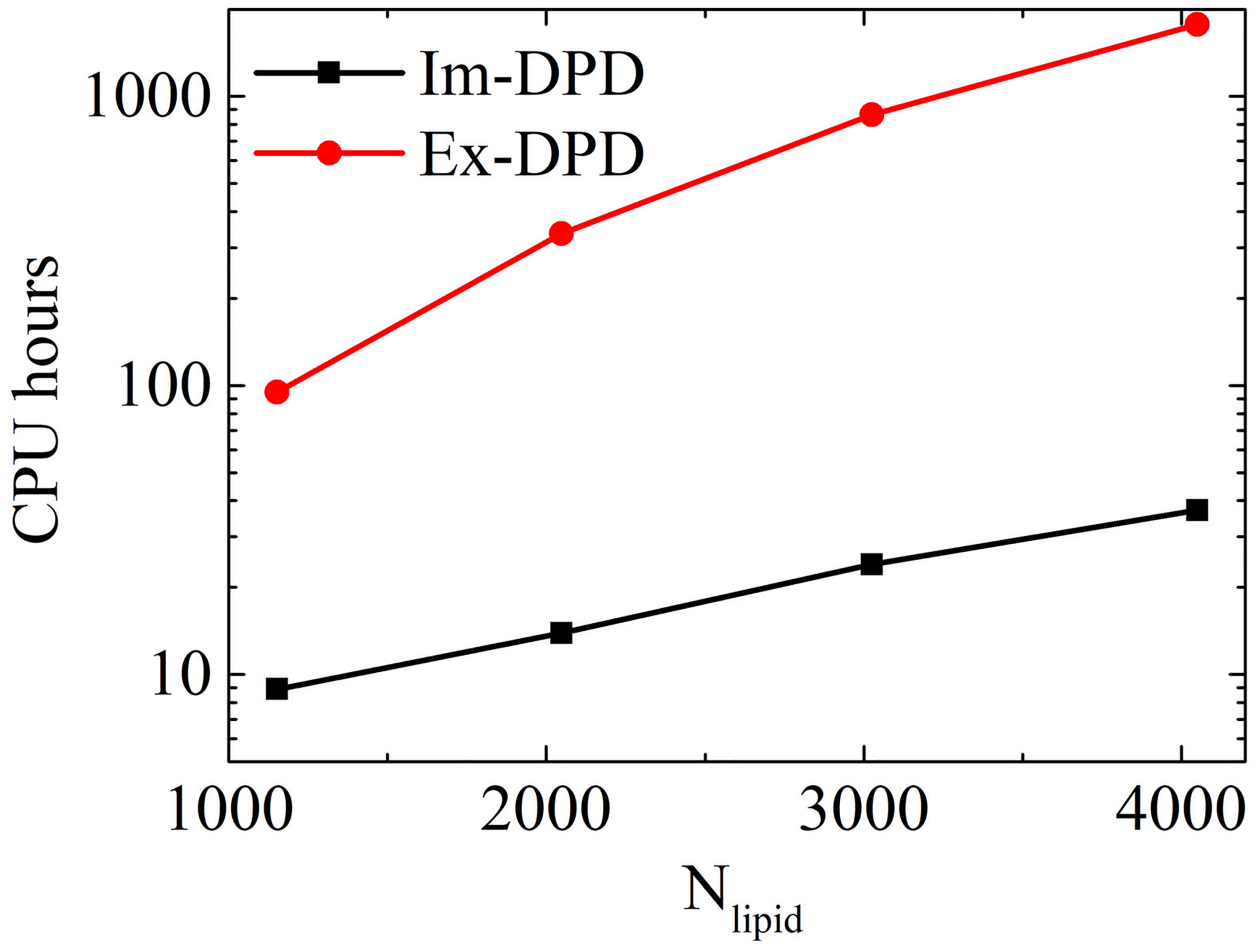
CPU hours to assemble vesicles using Ex-DPD and Im-DPD.

To demonstrate the efficiency of Im-DPD, we simulated vesicle formation starting from planar bilayers composed of 1152, 2048, 3025, and 4096 lipids. The time evolution of small vesicle formation is shown in Fig 16. A planar pre-assembled bilayer quickly transformed into a circular plate-like bicelle to minimize its circumference, followed by bending, wrapping up, and closing into a vesicle. We noted several detached free lipids ‘flying’ in the simulation box (Fig 16B). This is a normal phenomenon in implicit-solvent CG simulations [62, 77]. These lipids followed a ballistic trajectory because of the low local particle density [62]. The bilayer of 1152 lipids required 8.89 CPU hours to assemble a vesicle using Im-DPD; it required 95 CPU hours using Ex-DPD. Around 90 percent of the simulation time was saved when the solvent degrees of freedom was omitted. More remarkably, for larger vesicles composed of 2048, 3025, or 4096 lipids, a 20-fold to 50-fold increase in speed was acquired (Fig 17), demonstrating the high efficiency of our Im-DPD force field.

### Limitations of Im-DPD

Traditional AAMD and CGMD simulations of lipid membranes employ the Lennard-Jones potential, which has a repulsive *r*^-12^ part to approximate the short-distance repulsion, as well as an attractive *r*^-6^ part to describe the van-der-Waals interaction. The Ex-DPD non-bonded interaction is purely repulsive, rendering too compact membranes, which is relieved in Im-DPD by introducing an extra long-range attractive term, as evidenced from the density profile and the free energy barriers (Fig 9, Tables 6 and 7). However, the mismatching between DPD and AAMD still exists. To further match them, Im-DPD parameters need to be obtained from AAMD, rather than from Ex-DPD simulations. Perhaps a thorough solution would be the development of a brand new Ex-DPD, that strikes the proper balance between the soft-bead DPD potential and the hard-core Lennard-Jones potential.

Another limitation arises from the criteria of parameterization, e.g., the elastic property for DPD, while the partition free energy for Martini. As a matter of fact, the preferred properties are well reproduced at the expense of weakly sacrificing other characters. For instance, the DMPC bending modulus estimated by Martini is two to three times (1.1~1.7 ×10^−19^ J) [26] larger than experimental value (0.56 ×10^−19^ J) [54] or DPD (0.4~0.5 ×10^−19^ J). On the contrary, DPD is a weak reproduction of AAMD, in comparison to the rather good correspondence between Martini and AAMD, in terms of the flip-flop energy consumption (Tables 6 and 7).

A common limitation in CGMD/DPD method is the unrealistic treatment of the conformational change of a (poly)peptide. Because of the losing atomistic information, a peptide cannot spontaneously and correctly rearrange its backbone to fit different circumstances, e.g., the membrane surface or the water environment. In the current version of DPD force field, a Morse potential is applied to mimic fixed secondary structures, as the dihedral potential is employed in Martini. Therefore, the considerable change of the peptide structure is not handled reasonably. To alleviate this problem, one could consider the elastic network model [78], or more precisely the polarized CG model [79].

## Conclusion

An efficient and reliable solvent-free DPD force field for phospholipids and polypeptides was developed based on a popular Martini CG mapping rule to improve transferability. Implicit DPD parameters were obtained from explicit DPD simulations combined with complementary refinements that yielded better performance. The validity of this Im-DPD force field was justified by investigating structural and elastic properties of lipid bilayer membranes, free energy profiles for lipid flip-flop and amino acid analogues translocating across the membrane, and pore formation induced by the AMPs magainin and melittin. Overall results showed good agreement with Ex-DPD simulations, atomistic MD simulations, and experimental measurements, providing a versatile and reliable application. Most significantly, when used to simulate a large vesicle composed of thousands of lipids, nearly 99 percent of the computation time was saved by implicitly processing the bulk solvent. Therefore, we expect a wide range of applications for Im-DPD that will bridge the gap between the atomistic and mesoscopic levels.
